# Effectiveness of nirsevimab among infants in their first RSV season in the United States, October 2023–March 2024: a test-negative design analysis

**DOI:** 10.1016/j.lana.2025.101196

**Published:** 2025-08-06

**Authors:** Amanda B. Payne, Steph Battan-Wraith, Elizabeth A.K. Rowley, Melissa S. Stockwell, Sara Y. Tartof, Kristin Dascomb, Stephanie A. Irving, Brian Dixon, Sarah W. Ball, Mark W. Tenforde, Gabriela Vazquez-Benitez, Ashley B. Stephens, Jungmi Han, Karthik Natarajan, S. Bianca Salas, Cassandra Bezi, Lina S. Sy, Bruno Lewin, Tamara Sheffield, Julie Arndorfer, Daniel Bride, Josh Van Otterloo, Allison L. Naleway, Padma D. Koppolu, Shaun Grannis, William Fadel, Colin Rogerson, Tom Duszynski, Sarah E. Reese, Patrick K. Mitchell, Sean Chickery, Heidi L. Moline, Morgan Najdowski, Allison Avrich Ciesla, Emily L. Reeves, Malini DeSilva, Katherine E. Fleming-Dutra, Ruth Link-Gelles

**Affiliations:** a National Center for Immunization and Respiratory Diseases, Centers for Disease Control and Prevention, Atlanta, GA, USA; b Westat, Rockville, MD, USA; c Columbia University Irving Medical Center, New York, NY, USA; d Kaiser Permanente Southern California, Pasadena, CA, USA; e Kaiser Permanente Bernard J Tyson School of Medicine, Pasadena, CA, USA; f Intermountain Health, Salt Lake City, UT, USA; g Kaiser Permanente Center for Health Research, Portland, OR, USA; h Center for Biomedical Informatics, Regenstrief Institute, Indianapolis, IN, USA; i Richard M. Fairbanks School of Public Health, Indiana University, Indianapolis, IN, USA; j HealthPartners Institute, Minneapolis, MN, USA; k Indiana University School of Medicine, Indianapolis, IN, USA; l Eagle Health Analytics, San Antonio, TX, USA; m United States Public Health Service Commissioned Corps, Rockville, MD, USA

**Keywords:** Respiratory syncytial virus, Effectiveness, Immunization, Test negative design, Nirsevimab

## Abstract

**Background:**

In August 2023, the Centers for Disease Control and Prevention recommended nirsevimab, a long-acting monoclonal antibody, for all U.S. infants aged <8 months entering or born during their first respiratory syncytial virus (RSV) season. Our aim was to estimate nirsevimab effectiveness against RSV-associated emergency department (ED) encounters and hospitalisation among U.S. infants during the 2023–2024 RSV season.

**Methods:**

We conducted a test-negative analysis using electronic health record (EHR) data from 6 healthcare systems, including ED encounters and hospitalizations with a diagnosis of RSV-like illness (RLI) during October 8, 2023–March 31, 2024, among infants aged <8 months as of October 1, 2023, or born during the study period. Nirsevimab effectiveness was estimated by comparing children who received nirsevimab with those who did not among RSV-positive and RSV-negative encounters, adjusting for age, race and ethnicity, sex, calendar day, and geographic region and excluding infants whose mother received RSV vaccination during pregnancy.

**Findings:**

Among 5039 ED encounters with RLI among infants in their first RSV season, 2045 (41%) were RSV-positive and 446 (9%) received nirsevimab, with a median time since dose of 52 days (interquartile range [IQR]: 27–84 days). Among 1025 hospitalizations with RLI among infants in their first RSV season, 605 (59%) were RSV-positive and 95 (9%) received nirsevimab, with a median time since dose of 48 days (IQR: 24–82 days). Nirsevimab effectiveness was 77% (95% CI: 69%–83%) against RSV-associated ED encounters and 98% (95% CI: 95%–99%) against RSV-associated hospitalisation.

**Interpretation:**

Nirsevimab was effective in preventing RSV-associated ED encounters and hospitalisation among infants in their first RSV season, with greatest protection against hospitalisation. However, these estimates reflect a short interval from nirsevimab administration to RLI onset. Since nirsevimab is a passive immunization and concentration is expected to wane over time, it is important to continue monitoring effectiveness to assess effectiveness with increased time since dose.

**Funding:**

This work was supported by the 10.13039/100000030Centers for Disease Control and Prevention (contracts 75D30121D12779 to Westat and 75D30123C18039 to Kaiser Foundation Hospitals).

**Research in context**

**Evidence before this study**

Respiratory syncytial virus (RSV) causes substantial morbidity and mortality among infants. In 2023 widespread use of RSV immunizations, including nirsevimab, was recommended in the United States to prevent lower respiratory tract disease among infants born during or entering their first RSV season. We searched PubMed on December 1, 2024, using the search terms ((RSV) OR (respiratory syncytial)) AND (nirsevimab) AND (effectiveness) for all available literature regarding nirsevimab effectiveness among infants in their first RSV season published between August 3, 2023 (which was the date nirsevimab was recommended in the USA) and December 1, 2024, with no language restrictions. We found 17 relevant publications, with reported estimates of nirsevimab effectiveness against RSV-associated emergency department (ED) encounters ranging from 17% to 88% and estimates of nirsevimab effectiveness against RSV-associated hospitalization ranging from 70% to 94%. However, most nirsevimab effectiveness data were collected in Spain and France. There are limited data regarding nirsevimab effectiveness from the Americas. To confirm RSV immunization products are working, to inform future immunization policy decisions, and to increase immunization confidence, we sought to estimate effectiveness of nirsevimab among infants during the 2023–2024 respiratory virus season in the United States in the Virtual SARS-CoV-2, Influenza, and Other respiratory viruses Network (VISION).

**Added value of this study**

In this multisite analysis of hospitalizations and ED encounters identified in VISION from Oct 1, 2023, to March 31, 2024, among infants born during or entering their first RSV season who presented with RSV-like illness and were clinically tested for RSV, nirsevimab provided protection with effectiveness of 77% against RSV-associated ED encounters and 98% against RSV-associated hospitalization during the first RSV season after nirsevimab was recommended. These findings add to the limited data regarding niresevimab effectiveness against severe RSV disease in the United States and indicate that the immunization has the potential to reduce morbidity and mortality from severe RSV disease among infants.

**Implications of all the available evidence**

Nirsevimab was effective against RSV-associated hospitalization and ED encounters among infants during the first season after nirsevimab recommendation. These findings provide additional context regarding the benefit of RSV immunization among infants.

## Introduction

Each year, respiratory syncytial virus (RSV) leads to approximately 1.5 million outpatient visits, 520,000 emergency department (ED) visits, and 58,000–80,000 hospitalisations among children <5 years of age in the United States.^1^^,^^2^ Most children are infected with RSV during the first year of life.^3^ While RSV usually causes mild illness, it can lead to severe illness, especially in infants; RSV is the leading cause of hospitalisation among infants in the United States.^4^ Certain conditions, including prematurity (≤30 weeks’ gestation) and congenital lung and heart disease, are risk factors for severe illness; however, even healthy, full-term infants are at risk of severe RSV.^5^^,^^6^

Since 1998, palivizimab, a monoclonal antibody, has been licensed by the U.S. Food and Drug Administration (FDA) for prevention of severe RSV among infants and young children at increased risk of severe RSV.^7^ However, due to relatively high cost and requirement for monthly administration, palivizimab was only recommended for use among a small group of infants and young children with significant risk factors for severe RSV disease.^6^

In July 2023, the FDA approved nirsevimab (Beyfortus, Sanofi and AstraZeneca), a long-acting monoclonal antibody, for prevention of RSV-associated lower respiratory tract illness (LRTI) in infants.^8^ In August 2023, the Centers for Disease Control and Prevention’s (CDC) Advisory Committee on Immunization Practices (ACIP) recommended a single dose of nirsevimab for infants aged <8 months entering or born during their first RSV season, with administration generally recommended from October through March, and for young children aged 8–19 months at increased risk for severe RSV disease and entering their second RSV season.^9^ Nirsevimab can be administered in the birth hospital or during an outpatient encounter, such as a well child visit.^10^

In August 2023, the FDA approved maternal RSV vaccine (Abrysvo, Pfizer Inc.) for pregnant women during 32–36 weeks’ gestation for prevention of RSV-associated LRTI in infants aged <6 months.^11^ The vaccine was recommended by ACIP in September 2023 for seasonal use, meaning during September–January in most of the continental United States.^12^ Either maternal RSV vaccination during pregnancy or nirsevimab administration for the infant is recommended to prevent RSV-associated LRTI among infants, but both are not needed for most infants.^10^

In October 2023, CDC issued a Health Alert Network (HAN) advisory providing options to protect infants against RSV in the context of limited supply of nirsevimab, with priority given to infants at highest risk for severe RSV, including those <8 months with certain underlying conditions and infants age <6 months without those conditions.^13^ In January 2024, as nirsevimab supply increased, CDC communicated that providers should return to the ACIP recommendations.^14^

Nirsevimab was licensed based on clinical trial data.^15^ Real-world data on the effectiveness of nirsevimab in the United States are limited. These data are needed to confirm RSV immunization products are working, to inform future vaccine policy decisions, and to increase immunization confidence. We sought to estimate effectiveness of nirsevimab among infants during the 2023–2024 respiratory virus season in the United States.

## Methods

### Study population and data sources

The Virtual SARS-CoV-2, Influenza, and Other respiratory viruses Network (VISION) is an electronic health record (EHR)-based network in the United States.^16^ For paediatric product effectiveness (PE) analyses, data on emergency department (ED) encounters and hospitalisations from the following healthcare systems were included: Columbia University Irving Medical Center (CUIMC; New York), HealthPartners Institute (Minnesota and Wisconsin), Intermountain Health (Utah), Kaiser Permanente Southern California (KPSC), Kaiser Permanente Center for Health Research (KPCHR; Oregon and Washington), and Regenstrief Institute (Indiana). In total, encounter data were contributed by 127 EDs and 107 hospitals providing paediatric care.

In this test negative design (TND) investigation, ED encounters or hospitalisations with a diagnosis of RSV-like illness (RLI) and RSV testing occurring during October 8, 2023–March 31, 2024, among infants aged <8 months as of October 1, 2023, or born after that date were included. As the investigation design required testing, ED encounters and hospitalisations were included due to substantial rates of clinician-driven RSV testing in these settings. Dates of eligible encounters corresponded to a period of RSV circulation in most of the continental United States.^17^ RLI was identified based on presence of an International Classification of Diseases, 10th Revision (ICD-10) discharge diagnosis code corresponding to RLI in any position, a modification of a previously-used definition of acute respiratory illness (ARI)^18^ (Supplementary Table S1). ED encounters and hospitalisations were excluded for patients with potentially incomplete immunization or RSV testing records (e.g., those who were transferred from out of network for the RLI encounter, those who resided in a different state than the site, and encounters with insufficient lag time to update immunization data sources, according to each site’s immunization data reporting policies). ED encounters and hospitalisations were included if RSV test results from specimens collected 10 days prior to 72 h after the date of the ED encounter or hospital admission were available. Hospitalisations lasting <24 h were excluded. Hospital readmissions within 30 days were collapsed into a single event, and ED encounters occurring within 7 days were collapsed into a single event.

### Study definitions

The index date for each encounter was defined as either the date of collection of a respiratory specimen associated with the most recent positive or negative RSV test result before the encounter or the date of the encounter (if testing occurred on or after the encounter date). Test-positive cases were RLI-associated ED encounters or hospitalisations with a positive RSV molecular or antigen test, excluding ED encounters and hospitalisations also testing positive for SARS-CoV-2 or influenza during the 10 days prior to 72 h after the index date for the encounter (i.e., excluding known co-detections with RSV and either SARS-CoV-2 or influenza due to inability to determine if RSV or another co-infecting virus was causing RLI in these cases). Test-negative controls were RLI-associated ED encounters or hospitalisations with a negative RSV molecular test, regardless of SARS-CoV-2 or influenza test result. Encounters with only a negative RSV antigen test and no molecular test performed or only indeterminate RSV test results were excluded.

The exposure of interest was nirsevimab immunization, defined as receipt of nirsevimab 7 days or more prior to the index date for the RSV-associated ED encounter or hospitalisation, to exclude infants who received nirsevimab after being infected with RSV. Infant and maternal RSV immunization statuses were documented by EHRs, state and city registries, and insurance claims data (for a subset of sites). ED encounters or hospitalisations were excluded if the immunization history was implausible, i.e., if the nirsevimab dose was administered before October 1, 2023, as nirsevimab was not widely available in the United States prior to this date, or if > 1 dose of nirsevimab was received prior to the encounter. Additionally, to exclude ED encounters and hospitalisations for which the RLI may have started prior to nirsevimab receipt, ED encounters and hospitalisations were excluded if the nirsevimab dose was administered 0–6 days prior to the encounter and, relatedly, if the infant was aged <7 days. To address bias potentially introduced by including infants protected by a different RSV preventive product, ED encounters and hospitalisations among infants with evidence of maternal RSV vaccination or palivizumab use were excluded. Maternal RSV vaccination status was ascertained by linking maternal and infant records using site-specific methods (Supplementary Table S2). Additionally, ED encounters and hospitalisations among infants born after September 22, 2023, were excluded if ascertainment of maternal RSV vaccination status for that infant was not possible.

Infant age, race and ethnicity, sex, social vulnerability index (SVI) of residence,^19^ date of ED encounter or hospitalisation, geographic region, Medicaid status, facility urban-rural classification,^20^ and underlying medical conditions were collected from the EHR. Underlying medical conditions were defined based on ICD-10 discharge diagnosis codes recorded at the time of the ED encounter or hospitalisation (Supplementary Table S3). A subset of underlying medical conditions, including chronic lung disease of prematurity, congenital heart disease, Down Syndrome, neurological and/or musculoskeletal conditions, cystic fibrosis, congenital airway abnormality, and reactive airway disease, were considered conditions conferring increased risk of severe RSV.^6^ Additional variables considered in subgroup and sensitivity analyses (e.g., preterm birth status, neonatal intensive care unit [NICU] admission at time of birth, and birthweight) were also collected from the EHR (Supplementary Methods S1).

### Statistical analysis

In univariate comparisons of test-positive cases and test-negative controls and those with and without documented nirsevimab immunization, a standardized mean difference >0.2 was considered a meaningful difference in the distribution of a variable between groups.^21^ Nirsevimab effectiveness was estimated by comparing children who received nirsevimab with those who did not among RSV-positive and RSV-negative encounters, with nirsevimab effectiveness being calculated as one minus the adjusted odds ratio. The adjusted odds ratio was estimated using multivariable logistic regression, adjusting for age, race and ethnicity, sex, calendar day (days since Oct 8, 2023), and geographic region a priori. We evaluated whether additional adjustment of other covariates with SMD >0.2 in univariate analyses impacted PE estimates by separately including these covariates in the model and comparing resulting PE estimates to those from models that did not include the covariate. Nirsevimab effectiveness was estimated overall and by time since dose. For strata with very small sample sizes, Firth’s penalized logistic regression method was used.^22^ A secondary analysis estimated the adjusted odds ratio using a weighted multivariable logistic regression with inverse propensity-to-be-immunized weights and covariate adjustment for age, race and ethnicity, sex, calendar day (days since October 1, 2023) and geographic region (Supplementary Methods S2 and Table S4).

Sub-group analyses evaluated nirsevimab effectiveness among infants born preterm and those with underlying medical conditions. Additionally, sensitivity analyses evaluated if any of the following changed effectiveness estimates: 1) varying the dates of eligible encounters based on RSV circulation and/or nirsevimab implementation at each site; 2) restricting cases to RSV molecular-positive; 3) excluding controls testing positive for SARS-CoV-2 or influenza; 4) including infants with evidence of nirsevimab receipt 0–6 days prior to the encounter; 5) excluding infants with immunocompromising conditions; 6) restricting to ARI; 7) excluding sites testing a relatively low proportion of encounters; and 8) varying the time since dose categories (Supplementary Table S5).

All analyses were conducted in SAS software, version 9.4 (SAS Institute) or R software, version 4.4.0 (R Foundation for Statistical Computing).

### Ethics approval

The activity was reviewed by CDC, deemed not research, and was conducted consistent with applicable federal law and CDC policy.^o^ The protocol was reviewed and approved as a research activity by one VISION site; the protocol was reviewed and deemed not research by all other VISION sites. Written consent was not required.

### Role of funding source

This work was supported by the U.S. Centers for Disease Control and Prevention (CDC) (contracts 75D30121D12779 to Westat and 75D30123C18039 to Kaiser Foundation Hospitals). CDC employees were involved in the design and conduct of the study; collection, management, analysis, and interpretation of the data; preparation, review, and approval of the manuscript; and decision to submit the manuscript for publication. The findings and conclusions in this report are those of the authors and do not necessarily represent the official position of the CDC.

## Results

### ED encounters for RLI among infants

**Fig. 1:**
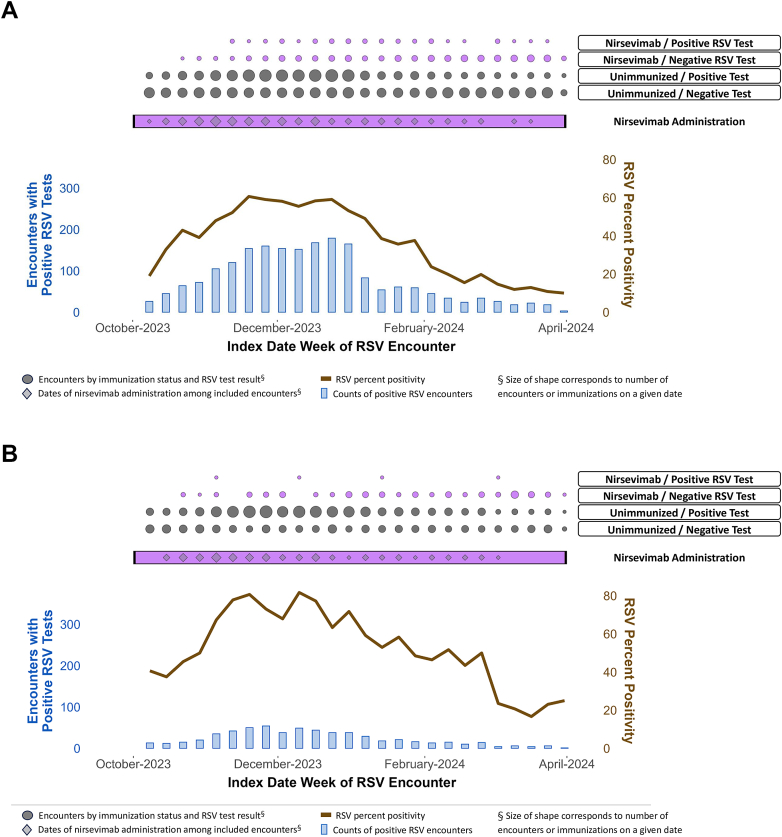
**Encounters for RSV-like illness among infants in their first RSV season, VISION, October 1, 2023–March 31, 2024.** A: Distribution of emergency department encounters for RSV-like illness among infants in their first RSV season, by vaccination and positivity status—October 2023–March 2024. B: Distribution of hospitalizations for RSV-like illness among infants in their first RSV season, by immunization and RSV positivity status—October 2023–March 2024. RSV denotes respiratory syncytial virus.

During October 8, 2023–March 31, 2024, we identified 5039 eligible ED encounters for RLI among infants in their first RSV season (aged <8 months as of or born after October 1, 2023) (Supplementary Fig. S1). RSV positivity peaked during late December 2023, and nirsevimab was administered throughout the observation period (Fig. 1A). Among the ED encounters for RLI, 2045 (41%) were RSV-positive and 446 (9%) had received nirsevimab ≥7 days prior to the encounter (Table 1). RSV-positivity status varied by site, month of encounter, age, Medicaid status, and SVI. Nirsevimab immunization status varied by site, facility urban-rural classification, month of encounter, age, race and ethnicity, Medicaid status, and maternal/infant linkage status.

**Table 1:** Characteristics of emergency department encounters for RSV-like illness by RSV test result and nirsevimab immunization status, VISION Network, Oct 8, 2023–March 31, 2024.

Characteristic	All ED encounters, N (column %)	RSV test result^a^, N (column %)		SMD^b^	Nirsevimab status^c^, N (row %)		SMD^d^
		Negative	Positive		No nirsevimab	Received nirsevimab	
All ED encounters, N	5039	2994	2045		4593	446	
Site							
A	280 (6)	184 (6)	96 (5)	0.37	249 (89)	31 (11)	0.51
B	524 (10)	342 (11)	182 (9)		480 (92)	44 (8)	
C	808 (16)	351 (12)	457 (22)		707 (88)	101 (13)	
D	155 (3)	99 (3)	56 (3)		131 (85)	24 (15)	
E	2177 (43)	1240 (41)	937 (46)		2075 (95)	102 (5)	
F	1095 (22)	778 (26)	317 (16)		951 (87)	144 (13)	
Facility urban-rural classification							
Large central metro	2857 (57)	1658 (55)	1199 (59)	0.17	2573 (90)	284 (10)	0.22
Large fringe metro	804 (16)	503 (17)	301 (15)		733 (91)	71 (9)	
Medium metro	307 (6)	210 (7)	97 (5)		288 (94)	19 (6)	
Small metro	527 (11)	335 (11)	192 (9)		496 (94)	31 (6)	
Micropolitan	166 (3)	96 (3)	70 (3)		151 (91)	15 (9)	
Non-core	165 (3)	90 (3)	75 (4)		158 (96)	7 (4)	
Unknown	213 (4)	102 (3)	111 (5)		194 (91)	19 (9)	
Month of encounter^e^							
October 2023	500 (10)	338 (11)	162 (8)	0.75	498 (100)	2 (0)	0.81
November 2023	996 (20)	465 (16)	531 (26)		968 (97)	28 (3)	
December 2023	1255 (25)	529 (18)	726 (36)		1165 (93)	90 (7)	
January 2024	842 (17)	466 (16)	376 (18)		753 (89)	89 (11)	
February 2024	693 (14)	539 (18)	154 (8)		592 (85)	101 (15)	
March 2024	748 (15)	652 (22)	96 (5)		612 (82)	136 (18)	
April 2024	5 (<1)	5 (<1)	0 (<1)		5 (100)	0 (0)	
Age (months), median [IQR]	5 [3, 8]	6 [3, 8]	5 [2, 7]	0.23	5 [3, 8]	3 [1, 6]	0.57
Born before or on October 1, 2023	3926 (78)	2300 (77)	1626 (80)	0.07	3741 (95)	185 (5)	0.90
Born after October 1, 2023	1113 (22)	694 (23)	419 (20)		852 (77)	261 (23)	
Female	2221 (44)	1293 (43)	928 (45)	0.04	2046 (92)	175 (8)	0.11
Race and ethnicity^f^							
Black or African American, non-Hispanic	611 (12)	395 (13)	216 (11)	0.14	569 (93)	42 (7)	0.29
White, non-Hispanic	1989 (39)	1106 (37)	883 (43)		1857 (93)	132 (7)	
Hispanic or Latino	1855 (37)	1149 (38)	706 (35)		1646 (89)	209 (11)	
Other, non-Hispanic	345 (7)	204 (7)	141 (7)		312 (90)	33 (10)	
Unknown	239 (5)	140 (5)	99 (5)		209 (87)	30 (13)	
Social Vulnerability Index of residence^g^							
Quartile 1	770 (15)	421 (14)	349 (17)	0.21	714 (93)	56 (7)	0.17
Quartile 2	853 (17)	490 (16)	363 (18)		777 (91)	76 (9)	
Quartile 3	720 (14)	405 (14)	315 (15)		665 (92)	55 (8)	
Quartile 4	776 (15)	419 (14)	357 (18)		718 (93)	58 (7)	
Unknown	1920 (38)	1259 (42)	661 (32)		1719 (90)	201 (10)	
Medicaid coverage^h^	3015 (60)	2021 (68)	994 (49)	0.45	2743 (91)	272 (9)	0.21
Linkage to maternal RSV vaccination records^i^	4361 (87)	2561 (86)	1800 (88)	0.07	3934 (90)	427 (10)	0.35
No. of UMC categories^j^							
0	4861 (97)	2870 (96)	1991 (97)	0.10	4444 (91)	417 (9)	0.18
1	158 (3)	107 (4)	51 (3)		136 (86)	22 (14)	
2	18 (<1)	15 (<1)	3 (<1)		11 (61)	7 (39)	
≥3	2 (<1)	2 (<1)	0 (0)		2 (100)	0 (0)	
Presence of UMC category							
Respiratory disease	58 (1)	37 (1)	21 (1)	0.02	49 (84)	9 (16)	0.08
Non-respiratory disease	170 (3)	118 (4)	52 (3)	0.08	138 (81)	32 (19)	0.19
≥1 high-risk UMC^k^	121 (2)	80 (3)	41 (2)	0.04	101 (83)	20 (17)	0.13
Chronic lung disease of prematurity	3 (<1)	3 (<1)	0 (0)	0.05	0 (0)	3 (100)	0.12
Congenital heart disease	42 (1)	29 (1)	13 (1)	0.04	34 (81)	8 (19)	0.09
Down syndrome	9 (<1)	7 (<1)	2 (<1)	0.03	7 (78)	2 (22)	0.05
Neuromuscular disease	47 (1)	33 (1)	14 (1)	0.04	38 (81)	9 (19)	0.10
Cystic fibrosis	0 (0)	0 (0)	0 (0)	<0.01	0 (0)	0 (0)	–
Congenital airway abnormality	9 (<1)	7 (<1)	2 (<1)	0.03	7 (78)	2 (22)	0.05
Reactive airway disease	32 (1)	19 (1)	13 (1)	<0.01	30 (94)	2 (6)	0.03
Preterm birth^l^	429 (9)	291 (10)	138 (7)	0.11	359 (84)	70 (16)	0.25
Nirsevimab immunization	446 (9)	383 (13)	63 (3)	0.37	–	446 (100)	–
Dose 7–59 days prior to encounter	250 (5)	203 (7)	47 (2)	0.37	–	250 (100)	–
Dose 60–164 days prior to encounter	196 (4)	180 (6)	16 (1)		–	196 (100)	–
Age at immunization (days), median, [IQR]	30 [2, 124]	32 [3, 125]	19 [2, 51]	0.34	–	30 [2, 124]	–

RSV = respiratory syncytial virus; ED = emergency department; SMD = standardized mean difference; IQR = interquartile range; UMC = underlying medical condition; VISION = Virtual SARS-CoV-2, Influenza, and Other respiratory viruses Network.

a RSV test results from tests conducted within 10 days prior to 3 days after date of ED encounter. If multiple tests were conducted, positive results were prioritized over negative results, then the most proximal test to the date of the encounter, and then viral culture test results over molecular assay test results, molecular assay test results over rapid molecular assay test results, rapid molecular assay test results over rapid antigen test results, rapid antigen test results over fluorescent antibody test results, and fluorescent antibody test results over serology test results. One site did not capture fluorescent antibody or serology test results. Test results for all viral culture, molecular, and rapid molecular tests and positive rapid antigen tests were included. Test results for negative rapid antigen tests and fluorescent antibody and serology tests were not included.

b The standardized mean difference is the difference between the distribution of RSV-positive encounters and the distribution of RSV-negative encounters. A standardized mean difference >0.2 was considered a meaningful difference between groups.

c Encounters were excluded if the infant received >1 nirsevimab dose; received nirsevimab prior to October 1, 2023; received nirsevimab 0–6 days prior to the index date; received another RSV immunization product, or the infant was aged <7 days at the time of the encounter.

d The standardized mean difference is the difference between the distribution of unimmunized encounters and the distribution of immunized encounters. A standardized mean difference >0.2 was considered a meaningful difference between groups.

e The index date for each encounter was defined as either the date of collection of a respiratory specimen associated with the most recent positive or negative RSV test result before the encounter or the date of the encounter (if testing occurred only after the encounter date). Thus, some ED visits may have occurred after March 31, 2024.

f “Non-Hispanic, Other” race includes persons reporting non-Hispanic ethnicity and any of the following for race: American Indian or Alaska Native, Asian, Native Hawaiian or other Pacific Islander, other races not listed, and multiple races; because of small numbers, these categories were combined. “Unknown” includes persons with missing race and ethnicity in their electronic health records.

g Reflects the quartiles of the distribution of Social Vulnerability Index (SVI) among encounters included in this analysis. SVI is defined based on the census tract of residence. The CDC/ATSDR SVI uses 16 U.S. census variables to determine social vulnerability for each census tract. Higher SVI values correspond to higher social vulnerability, which refers to the potential negative effects on communities caused by external stresses on human health.

h N = 361 with unknown Medicaid coverage status as of encounter date.

i Linkage to maternal RSV vaccination records was required for inclusion for all infants born on or before September 22, 2023.

j UMC categories included pulmonary, cardiovascular, cerebrovascular, musculoskeletal, neurologic, hematologic, endocrine, renal, and gastrointestinal.

k High-risk conditions included International Classification of Disease, 10th Revision (ICD-10) discharge diagnosis codes for: chronic lung disease of prematurity, congenital heart disease, Down Syndrome, neurological and/or musculoskeletal conditions, cystic fibrosis, congenital airway abnormality, and reactive airway disease.

l Preterm birth status was derived from ICD-10 discharge diagnosis codes and infant records. If either an ICD-10 code corresponding to preterm birth (P07.∗) was listed among the discharge diagnoses as the time of the RLI encounter or the recorded gestational age at birth was <37 weeks, then the infant was considered to have been born preterm. Gestational age at birth was unknown for 2230 (44%) of RLI ED encounters; for these encounters, preterm birth status was defined based on ICD-10 discharge diagnosis codes.

### Nirsevimab effectiveness against RSV-associated ED encounters among infants

Among 5039 ED encounters for RLI among infants in their first RSV season, 446 had evidence of nirsevimab receipt ≥7 days prior to the encounter, with a median time since dose of 52 days (interquartile range [IQR]: 27–84) (Table 2). RSV percent positivity was lower among those that had evidence of nirsevimab receipt compared with those without (14% vs. 43%), and the adjusted estimate of nirsevimab effectiveness against RSV-associated ED encounters was 77% (95% CI: 69%–83%) (Table 2). Effectiveness estimates were similar when assessed at 7–59 days and 60–164 days since immunization (Table 2). Estimates of nirsevimab effectiveness against RSV-associated ED encounters among infants recorded as born preterm and those with recorded underlying medical conditions were also similar, with CIs around the subgroup estimates overlapping that of the overall estimate (Supplementary Table S6). Additionally, estimates were similar in sensitivity and secondary analyses (Supplementary Tables S7 and S8).

**Table 2:** Nirsevimab effectiveness against RSV-associated ED encounters among infants in their first RSV season, VISION Network, Oct 8, 2023–March 31, 2024.

Nirsevimab dosage pattern | time since dose	Total ED encounters^a^	RSV-positive ED encounters N (Row %)	Median days since dose (IQR)	Adjusted PE (95% CI)^b^
No nirsevimab doses	4593	1982 (43)	N/A	ref
Nirsevimab, 7–164 days prior	446	63 (14)	52 (27–84)	77 (69–83)
7–59 days prior	250	47 (19)	29 (18–43)	76 (66–83)
60–164 days prior	196	16 (8)	90 (72–111)	78 (62–87)

RSV = respiratory syncytial virus; ED = emergency department; IQR = interquartile range; CI = confidence interval; Ref = reference group; PE = product effectiveness; VISION = Virtual SARS-CoV-2, Influenza, and Other respiratory viruses Network.

a ED encounters included those among infants in their first RSV season with a diagnosis of RSV-like illness (RLI), excluding infants with evidence of maternal RSV vaccination and infants who received nirsevimab <7 days prior to the index date for the encounter. RLI was defined as ≥1 International Classification of Disease 10th Revision discharge diagnosis code corresponding to one or more of the following: COVID-19 pneumonia, influenza pneumonia, other viral pneumonia, influenza disease, bacterial pneumonia, acute respiratory distress syndrome, asthma exacerbation, respiratory failure, other acute lower respiratory tract infection, sinusitis, acute upper respiratory tract infections, acute respiratory illness signs and symptoms, viral illness not otherwise specified, sepsis, respiratory failure, irritable/fussy infant, respiratory distress of newborn, congenital pneumonia, interstitial emphysema and related conditions, other respiratory conditions originating in the perinatal period, congenital viral diseases, bacterial sepsis of newborn, or other infections specific to the perinatal period.

b PE was calculated as (1—adjusted odds ratio) x 100%, with adjusted odds ratio calculated using logistic regression, adjusting for age, race and ethnicity, sex, calendar day, and geographic region.

### Hospitalisations with RLI among infants

During October 8, 2023–March 31, 2024, we identified 1025 eligible hospitalisations for RLI among infants in their first RSV season (Supplementary Fig. S2). Similar to ED encounters for RLI, RSV positivity peaked during late December 2023 among hospitalisations with RLI, and nirsevimab was administered throughout the observation period (Fig. 1B). Among hospitalisations with RLI, 605 (59%) were RSV-positive and 95 (9%) had received nirsevimab ≥7 days prior to the index date for the hospitalisation (Table 3). RSV-positivity status and nirsevimab immunization status varied by site, facility urban-rural classification, month of encounter, age, SVI, and presence of underlying medical conditions. Additionally, nirsevimab immunization status varied by race and ethnicity.

**Table 3:** Characteristics of hospitalizations with RSV-like illness by RSV test result and nirsevimab immunization status, VISION Network, Oct 8, 2023–March 31, 2024.

Characteristic	All Hospitalizations, N (column %)	RSV Test Result^a^, N (column %)		SMD^b^	Nirsevimab Status^c^, N (row %)		SMD^d^
		Negative	Positive		No Nirsevimab	Received Nirsevimab	
All hospitalizations, N	1025	420	605		930	95	
Site							
A	16 (2)	5 (1)	11 (2)	0.29	14 (88)	2 (12)	0.63
B	155 (15)	77 (18)	78 (13)		143 (92)	12 (8)	
C	284 (28)	113 (27)	171 (28)		246 (87)	38 (13)	
D	15 (1)	8 (2)	7 (1)		10 (67)	5 (33)	
E	297 (29)	95 (23)	202 (33)		288 (97)	9 (3)	
F	258 (25)	122 (29)	136 (22)		229 (89)	29 (11)	
Facility urban-rural classification							
Large central metro	710 (69)	295 (70)	415 (69)	0.25	641 (90)	69 (10)	0.32
Large fringe metro	150 (15)	59 (14)	91 (15)		133 (89)	17 (11)	
Medium metro	75 (7)	38 (9)	37 (6)		73 (97)	2 (3)	
Small metro	53 (5)	21 (5)	32 (5)		48 (91)	5 (9)	
Micropolitan	6 (1)	2 (<1)	4 (1)		5 (83)	1 (17)	
Non-core	1 (<1)	1 (<1)	0 (0)		1 (100)	0 (0)	
Unknown	30 (3)	4 (1)	26 (4)		29 (97)	1 (3)	
Month of encounter^e^							
October 2023	107 (10)	61 (15)	46 (8)	0.69	105 (98)	2 (2)	0.90
November 2023	247 (24)	70 (17)	177 (29)		236 (96)	11 (4)	
December 2023	257 (25)	70 (17)	187 (31)		245 (95)	12 (5)	
January 2024	190 (19)	75 (18)	115 (19)		167 (88)	23 (12)	
February 2024	114 (11)	60 (14)	54 (9)		97 (85)	17 (15)	
March 2024	106 (10)	81 (19)	25 (4)		76 (72)	30 (28)	
April 2024	4 (<1)	3 (1)	1 (<1)		4 (100)	0 (0)	
ICU admission^f^	240 (23)	104 (25)	136 (22)	0.12	215 (90)	25 (10)	0.08
IMV^g^	87 (8)	49 (12)	38 (6)	0.21	74 (85)	13 (15)	0.36
In-hospital death	7 (1)	7 (2)	0 (0)	0.18	4 (57)	3 (43)	0.21
Age (months), median [IQR]	4 [1, 7]	4 [1, 7]	3 [1, 6]	0.17	4 [1, 7]	3 [1, 6]	0.16
Born before or on October 1, 2023	694 (68)	275 (66)	419 (69)	0.08	649 (94)	45 (6)	0.47
Born after October 1, 2023	331 (32)	145 (35)	186 (31)		281 (85)	50 (15)	
Female	434 (42)	162 (39)	272 (45)	0.13	398 (92)	36 (8)	0.10
Race and ethnicity^h^							
Black or African American, non-Hispanic	77 (8)	28 (7)	49 (8)	0.19	70 (91)	7 (9)	0.41
White, non-Hispanic	436 (43)	168 (40)	268 (44)		411 (94)	25 (6)	
Hispanic or Latino	398 (39)	165 (39)	233 (39)		352 (88)	46 (12)	
Other, non-Hispanic	77 (8)	43 (10)	34 (6)		64 (83)	13 (17)	
Unknown	37 (4)	16 (4)	21 (3)		33 (89)	4 (11)	
Social Vulnerability Index of residence^i^							
Quartile 1	140 (14)	44 (10)	96 (16)	0.26	135 (96)	5 (4)	0.34
Quartile 2	136 (13)	60 (14)	76 (13)		122 (90)	14 (10)	
Quartile 3	142 (14)	50 (12)	92 (15)		131 (92)	11 (8)	
Quartile 4	157 (15)	55 (13)	102 (17)		142 (90)	15 (10)	
Unknown	450 (44)	211 (50)	239 (40)		400 (89)	50 (11)	
Medicaid coverage^j^	508 (50)	216 (51)	292 (48)	0.07	463 (91)	45 (9)	0.12
Linkage to maternal RSV vaccination^k^ records^m^	886 (86)	354 (84)	532 (88)	0.11	799 (90)	87 (10)	0.18
No. of UMC categories^l^							
0	750 (73)	240 (57)	510 (84)	0.66	701 (93)	49 (7)	0.54
1	167 (16)	97 (23)	70 (12)		145 (87)	22 (13)	
2	66 (6)	47 (11)	19 (3)		50 (76)	16 (24)	
≥3	42 (4)	36 (9)	6 (1)		34 (81)	8 (19)	
Presence of UMC category							
Respiratory disease	121 (12)	78 (19)	43 (7)	0.35	98 (81)	23 (19)	0.37
Non-respiratory disease	369 (36)	198 (47)	171 (28)	0.40	320 (87)	49 (13)	0.35
≥1 high-risk UMC^m^	227 (22)	150 (36)	77 (13)	0.56	186 (82)	41 (18)	0.51
Chronic lung disease of prematurity	19 (2)	16 (4)	3 (1)	0.23	14 (74)	5 (26)	0.21
Congenital heart disease	123 (12)	87 (21)	36 (6)	0.45	94 (76)	29 (24)	0.53
Down syndrome	16 (2)	15 (4)	1 (<1)	0.25	15 (94)	1 (6)	0.05
Neuromuscular disease	97 (10)	73 (17)	24 (4)	0.45	81 (84)	16 (16)	0.25
Cystic fibrosis	3 (<1)	3 (1)	0 (0)	0.12	1 (33)	2 (67)	0.19
Congenital airway abnormality	32 (3)	22 (5)	10 (2)	0.20	27 (84)	5 (16)	0.12
Reactive airway disease	38 (4)	15 (4)	23 (4)	0.01	34 (89)	4 (11)	0.03
Preterm birth^n^	145 (14)	83 (20)	62 (10)	0.27	120 (83)	25 (17)	0.34
Nirsevimab immunization	95 (9)	91 (22)	4 (1)	0.71	–	95 (100)	–
Dose 7–59 days prior to encounter	57 (6)	53 (13)	4 (1)	0.71	–	57 (100)	–
Dose 60–145 days prior to encounter	38 (4)	38 (9)	0 (0)		–	38 (100)	–
Age at immunization (days), median, [IQR]	34 [3, 124]	34 [3, 124]	33 [4, 73]	0.39	–	34 [3, 124]	–

RSV = respiratory syncytial virus; ED = emergency department; SMD = standardized mean difference; ICU = intensive care unit; IMV = invasive mechanical ventilation; IQR = interquartile range; UMC = underlying medical condition; VISION = Virtual SARS-CoV-2, Influenza, and Other respiratory viruses Network.

a RSV test results from tests conducted within 10 days prior to 3 days after date of hospital admission. If multiple tests were conducted, positive results were prioritized over negative results, then the most proximal test to the date of the encounter, and then viral culture test results over molecular assay test results, molecular assay test results over rapid molecular assay test results, rapid molecular assay test results over rapid antigen test results, rapid antigen test results over fluorescent antibody test results, and fluorescent antibody test results over serology test results. One site did not capture fluorescent antibody or serology test results. Test results for all viral culture, molecular, and rapid molecular tests and positive rapid antigen tests were included. Test results for negative rapid antigen tests and fluorescent antibody and serology tests were not included.

b The standardized mean difference is the difference between the distribution of RSV-positive encounters and the distribution of RSV-negative encounters. A standardized mean difference >0.2 was considered a meaningful difference between groups.

c Encounters were excluded if the infant received >1 nirsevimab dose; received nirsevimab prior to October 1, 2023; received nirsevimab 0–6 days prior to the index date; received another RSV immunization product, or the infant was aged <7 days at the time of the encounter.

d The standardized mean difference is the difference between the distribution of unimmunized encounters and the distribution of immunized encounters. A standardized mean difference >0.2 was considered a meaningful difference between groups.

e The index date for each encounter was defined as either the date of collection of a respiratory specimen associated with the most recent positive or negative RSV test result before the encounter or the date of the encounter (if testing occurred only after the encounter date). Thus, some hospital admissions may have occurred after March 31, 2024.

f N = 15 missing ICU status.

g N = 129 missing IMV status.

h “Non-Hispanic, Other” race includes persons reporting non-Hispanic ethnicity and any of the following for race: American Indian or Alaska Native, Asian, Native Hawaiian or other Pacific Islander, other races not listed, and multiple races; because of small numbers, these categories were combined. “Unknown” includes persons with missing race and ethnicity in their electronic health records.

i Reflects the quartiles of the distribution of Social Vulnerability Index (SVI) among encounters included in this analysis. SVI is defined based on the census tract of residence. The CDC/ATSDR SVI uses 16 U.S. census variables to determine social vulnerability for each census tract. Higher SVI values correspond to higher social vulnerability, which refers to the potential negative effects on communities caused by external stresses on human health.

j N = 23 with unknown Medicaid coverage status as of encounter date.

k Linkage to maternal RSV vaccination records was required for inclusion for all infants born on or before September 22, 2023.

l UMC categories included pulmonary, cardiovascular, cerebrovascular, musculoskeletal, neurologic, hematologic, endocrine, renal, and gastrointestinal.

m High-risk conditions included International Classification of Disease, 10th Revision (ICD-10) discharge diagnosis codes for: chronic lung disease of prematurity, congenital heart disease, Down Syndrome, neurological and/or musculoskeletal conditions, cystic fibrosis, congenital airway abnormality, and reactive airway disease.

n Preterm birth status was derived from ICD-10 discharge diagnosis codes and infant records. If either an ICD-10 code corresponding to preterm birth (P07.∗) was listed among the discharge diagnoses as the time of the RLI encounter or the recorded gestational age at birth was <37 weeks, then the infant was considered to have been born preterm. Gestational age at birth was unknown for 381 (37%) of RLI hospitalizations.

### Nirsevimab effectiveness against RSV-associated hospitalisation among infants

Among 1025 hospitalisations with RLI among infants in their first RSV season, 95 had evidence of nirsevimab receipt ≥7 days prior to the index date for the hospitalisation, with a median time since dose of 48 days (IQR: 24–82) (Table 4). RSV percent positivity was lower among those who had evidence of nirsevimab receipt compared with those without (4% vs. 65%), and the adjusted estimate of product effectiveness against RSV-associated hospitalisation was 98% (95% CI: 95%–99%) (Table 4). Similar results were seen in subgroup analyses among infants recorded as born preterm and among those with recorded underlying medical conditions (Supplementary Table S9) and in sensitivity and secondary analyses evaluating the impact of various analytic decisions (Supplementary Tables S10 and S11).

**Table 4:** Nirsevimab effectiveness against RSV-associated hospitalization among infants in their first RSV season, VISION Network, Oct 8, 2023–March 31, 2024.

Nirsevimab dosage pattern | time since dose	Total RLI hospitalizations^a^	RSV-positive RLI hospitalizations N (Row %)	Median days since dose (IQR)	Adjusted PE (95% CI)^b^
No nirsevimab doses	930	601 (65)	N/A	ref
Nirsevimab, 7–145 days prior	95	4 (4)	48 (24–82)	98 (95–99)

RSV = respiratory syncytial virus; ED = emergency department; IQR = interquartile range; CI = confidence interval; Ref = reference group; PE = product effectiveness; VISION = Virtual SARS-CoV-2, Influenza, and Other respiratory viruses Network.

a Encounters included those among infants in their first RSV season with a diagnosis of RSV-like illness (RLI), excluding infants with evidence of maternal RSV vaccination and infants who received nirsevimab <7 days prior to the index date for the encounter. RLI was defined as ≥1 International Classification of Disease 10th Revision discharge diagnosis code corresponding to one or more of the following: COVID-19 pneumonia, influenza pneumonia, other viral pneumonia, influenza disease, bacterial pneumonia, acute respiratory distress syndrome, asthma exacerbation, respiratory failure, other acute lower respiratory tract infection, sinusitis, acute upper respiratory tract infections, acute respiratory illness signs and symptoms, viral illness not otherwise specified, sepsis, respiratory failure, irritable/fussy infant, respiratory distress of newborn, congenital pneumonia, interstitial emphysema and related conditions, other respiratory conditions originating in the perinatal period, congenital viral diseases, bacterial sepsis of newborn, or other infections specific to the perinatal period.

b PE was calculated as (1—adjusted odds ratio) x 100%, with adjusted odds ratio calculated using logistic regression, adjusting for age, race and ethnicity, sex, calendar day, and geographic region.

## Discussion

In this multisite investigation of nirsevimab effectiveness among infants in their first RSV season, we found that nirsevimab was 77% effective against RSV-associated ED encounters and 98% effective against RSV-associated hospitalisation during the first season of nirsevimab availability in the United States, though median time since dosing was only 48–54 days. Our findings are among the first to demonstrate real-world nirsevimab effectiveness against RSV-associated ED encounters and hospitalisations in the United States.

Previously published data regarding nirsevimab effectiveness among infants in their first RSV season, largely from Spain and France but also from the New Vaccine Surveillance Network (NVSN) the Yale New Haven Health System, and Alaska’s Yukon-Kuskokwim region in the United States, have shown nirsevimab to be effective against RSV-associated ED encounters and hospitalisation, with estimates of effectiveness against RSV-associated ED encounters ranging from 55% to 88% and estimates of effectiveness against RSV-associated hospitalisation ranging from 70% to 90%.^23^, ^24^, ^25^, ^26^, ^27^, ^28^, ^29^, ^30^, ^31^, ^32^, ^33^, ^34^, ^35^, ^36^ Nirsevimab effectiveness estimates from these studies skew lower than those in this report. There may be several explanations for this. First, while not reported for all studies, the median time since dose among nirsevimab recipients included in this investigation was likely less than other studies. Nirsevimab uptake in the United States during the 2023–2024 RSV season was hampered by limited product availability, especially early in the season.^13^ As of October 23, 2023, only 14% of infants aged <8 months had received nirsevimab.^37^ In comparison, over 80% of eligible infants had received nirsevimab by October 31, 2023, in regions of Spain and France that implemented nirsevimab during the 2023–2024 RSV season.^38^ Furthermore, only approximately 40% of infants aged <8 months had received nirsevimab by the end of the 2023–2024 RSV season in the United States.^37^ As nirsevimab concentration is expected to decline with more time since dose, with corresponding declines in effectiveness,^39^ it is possible the longer time since dose among nirsevimab recipients in European studies is reflected in lower effectiveness estimates compared with those in this report. Second, at least two previously-published studies included encounters occurring during periods of low RSV circulation, which might lead to lower effectiveness estimates.^35^^,^^36^ General principles for estimating product effectiveness indicate effectiveness should be measured under conditions where the immunized and unimmunized have equal chance of exposure to the pathogen of interest, which is generally when circulation of the pathogen is relatively high.^40^ However, our results were robust to changes in dates of eligible encounters, indicating this may not fully explain the difference noted. Third, this investigation relied on clinician-directed RSV testing, and it is possible infants included in this investigation reflect a more severe RSV-positive patient population due to the possibility that clinicians may be more likely to test infants with severe RSV disease.^41^ This hypothesis is supported by the observation of a higher effectiveness against more severe outcomes (e.g., RSV-associated hospitalisation) compared with less severe outcomes (e.g., RSV-associated ED encounters) seen in this report and in other nirsevimab effectiveness investigations.^26^, ^27^, ^28^ However, while estimated effectiveness against RSV-associated hospitalisation in this investigation (98% [95% CI: 95%–99%]) was slightly higher than that reported from NVSN (91% [95% CI: 79%–96%]),^34^ a similar proportion of RSV-positive encounters in this investigation (22%) were associated with ICU admission compared with infants enrolled in NVSN (23%), a U.S population-based, prospective surveillance platform for ARI, where respiratory specimens from enrolled children were collected and tested for RSV, regardless of clinician testing.^5^^,^^42^ Thus, the higher nirsevimab effectiveness estimates reported here cannot be fully explained, and continued monitoring of effectiveness with higher uptake, more time since dose, and in other surveillance platforms is warranted.

RSV is the leading cause of hospitalisation among infants in the United States.^4^ While RSV-associated hospitalisation rates among infants aged <1 year in the United States were lower during the 2023–2024 RSV season compared with the 2022–2023 RSV season, RSV-associated hospitalisation rates during the 2023–2024 RSV season were similar or higher to pre-COVD-19 pandemic RSV seasons.^5^^,^^43^ Despite the demonstrated real-world effectiveness of nirsevimab against severe RSV, there is no clear evidence of an impact on RSV-associated hospitalisation rates among infants during the 2023–2024 RSV season in the United States. This may be due to relatively low uptake of nirsevimab, especially early in the season, in the United States during the first season of availability.^37^ In comparison, in regions of Spain, where nirsevimab uptake among infants in their first RSV season was high, the administration of nirsevimab reduced RSV-associated hospitalisations among infants by approximately 75%.^44^ This highlights the potential impact of increased uptake of nirsevimab among infants in the United States during future RSV seasons and the importance of the timing of nirsevimab administration (ideally shortly before the RSV season or during the birth hospitalisation).

The strengths of this investigation include the large number of medical encounters in a geographically-diverse network of healthcare and research centres in the United States with integrated medical, laboratory, and immunization data. Integration of medical, laboratory, and immunization data allowed documentation of RSV test results obtained in a variety of settings within a time period around RLI encounters and documentation of immunizations administered in a variety of healthcare settings. Additionally, nirsevimab effectiveness estimates were robust in multiple sensitivity analyses, strengthening confidence in these estimates.

Our investigation of nirsevimab effectiveness among infants in their first RSV season is subject to at least six limitations. First, we cannot rule out the possibility that patients with RSV-positive encounters may have had an encounter for reasons other than RSV, which could have lowered nirsevimab effectiveness estimates. However, nirsevimab effectiveness estimates were similar in sensitivity analyses limiting encounters to those meeting the acute respiratory illness definition and in the sensitivity analyses excluding controls testing positive for SARS-CoV-2 or influenza. Second, because immunization registries, EHRs, and medical claims might not identify all nirsevimab, palivizumab, or maternal RSV doses administered, misclassification of RSV immunization status was possible. Under-ascertainment of RSV immunizations would likely result in underestimation of nirsevimab effectiveness.^45^, ^46^, ^47^ Third, nearly all RSV testing was clinician-directed, and there was site-to-site variability in RSV testing, including in use of antigen tests. Our main analysis included encounters testing positive for RSV via either an antigen or molecular test, and inclusion of antigen-positive cases could lead to misclassification of RSV status due to lower sensitivity and specificity of RSV antigen tests compared to molecular tests,^48^ which would likely underestimate nirsevimab effectiveness.^45^ However, we conducted a sensitivity analysis excluding encounters with positive antigen tests and noted little change in nirsevimab effectiveness estimates. Fourth, EHR data may not fully capture all underlying conditions, especially in the ED setting. Not accounting for presence of underlying medical conditions in the analysis could have resulted in confounding. Fifth, our analysis adjusted for relevant confounders, but residual confounding may be possible. During the first season of nirsevimab availability in the United States, a shortage of nirsevimab doses required prioritization of doses for those at highest risk of severe RSV,^13^ possibly introducing confounding by indication. Additionally, nirsevimab coverage in the United States during the 2023–2024 season was reported to vary by characteristics such as race/ethnicity and insurance type.^49^ However, sensitivity analyses estimated nirsevimab effectiveness using models that additionally included propensity-to-be-immunized weights based on a variety of potentially relevant confounders and noted little change in nirsevimab effectiveness estimates. Finally, the maximum time since nirsevimab dose prior to ED encounter or hospitalisation was 164 days in this analysis, with about half of encounters occurring within a month of nirsevimab receipt, and only 95 RLI hospitalisations among infants who received nirsevimab were identified. This limited the ability to evaluate waning effectiveness by time since dose.

Our analysis of medical encounters during the 2023–2024 RSV season showed nirsevimab was effective against RSV-associated ED encounters and hospitalisation among infants in their first RSV season during the first season after nirsevimab approval and recommendation. However, these estimates reflect a short interval from nirsevimab administration to RLI onset. Since nirsevimab is a passive immunization and concentration is expected to wane over time, it is important to continue monitoring effectiveness to assess effectiveness with increased time since dose. These findings add to the growing body of literature demonstrating the potential of broad nirsevimab uptake among infants to reduce severe RSV.

## Contributors

ABP and RLG conceptualized the assessment. ABP, RLG, and SBW drafted the statistical analysis plan. SBW, EAKR, SER, PKM and SC conceptualized and conducted the statistical analyses. ABP drafted the manuscript with input from HLM, RLG, and KFD. MSS, SYT, KD, SAI, BD, SWB, GVB, and MD were the site primary investigators. ABS, JH, KN, BS, CB, LSS, BL, TS, JA, DB, JVO, ALN, PDK, SJG, WFF, CR, and TD were site investigators. MN, AAC, and ELR managed and analyzed data and assisted with table and figure completion. RLG and MWT served as the CDC principal investigators for the VISION Network and ensured funding and site participation in the network. MSS, SYT, KD, SAI, BD, SWB, GVB, MD, ABS, JH, KN, SBS, CB, LSS, BL, TS, JA, DB, JVO, ALN, PDK, SJG, WFF, CR, and TD had access to their site’s raw data. ABP, SBW, EAKR, PKM, SC, MN, AAC, and ELR had access to and verified the data. ABP had final responsibility for the decision to submit the manuscript. All authors contributed to the interpretation of the findings and reviewed the final manuscript draft.

## Data sharing statement

Data collected for the study are not available. Data sharing agreements between CDC and VISION Network partner institutions prohibit CDC from making this dataset publicly available.

## Declaration of interests

SYT reports contracts from GSK and Pfizer. SYT, SBS, CB, LSS, and BL report institutional support for the VISION project via Contract # 75D30123C18039. MSS reports grants to their institution from CDC and National Institutes of Health and a paid role on the American Academy of Pediatrics, with payment made to their institution. BED, MBD, and WFF report an additional CDC contract for the Vaccine Safety Datalink. GVB reports grant funding from Sanofi for research unrelated to this work. ABS reports honoraria from the American Cancer Society for moderating a session at the New York State HPV Vaccination Summit and presenting for the HPV Vaccine ECHO series, from the University at Albany, State University of New York for a webinar on HPV Vaccine Communication Strategies for Patients and Communities, and from Bronxcare, Staten Island University Hospital for Grand Rounds presentations on HPV vaccine at multiple institutions, is a voluntary co-chair of the NY State HPV Coalition Health Equity Action Team, and a voluntary member of the NY State HPV Coalition Steering Committee. KN reports grants to their institution from National Institutes of Health National Cancer Institute, National Heart Lung, and Blood Institute, and Office of the Director. LSS reports grants to their institution from GSK, Moderna, and Dynavax. BL reports grant funding to their institution from the National Institutes of Health. TS reports unpaid participation as a member of the Advisory Committee on Immunization Practices Influenza Vaccine Work Group, chief of the Utah Adult Immunization Coalition, and as a member of the Utah Department of Health and Human Services Scientific Advisory Committee on Vaccines. SJG reports funding the National Institutes of Health’s National Center for Advancing Translation Sciences and National Institute of Mental Health. SBW, EAKR, SWB, SER, PKM, and SC report payments made to Westat via CDC Contract #75D30121D12779. MSS, SAI, BED, JH, KN, SJG, WFF, CR, and MBD report payments made to their institution by CDC via Westat. MBD reports funding from CDC as a Center for Excellence in Newcomer Health. All other authors declare no competing interests.

## Footnotes

o See e.g., 45 C.F.R. part 46.102(l)(2), 21 C.F.R. part 56; 42 U.S.C. §241(d); 5 U.S.C. §552a; 44 U.S.C. §3501 et seq.
