# Supplementary material for: Effectiveness of nirsevimab among infants in their first RSV season in the United States, October 2023–March 2024: a test-negative design analysis
Source: Lancet Reg Health Am. 2025 Aug 6;49:101196. doi: 10.1016/j.lana.2025.101196 (PMC12485534; doi:10.1016/j.lana.2025.101196)
Supplement: Supplementary Figs. S1 and S2 and Tables S1–S11 [file mmc1.pdf]

Title: Effectiveness of nirsevimab among infants in their first RSV season in the United States, October 2023 – March 2024

Authors:

Amanda Payne, PhD, MPH[1]; Steph Battan-Wraith, PhD[2]; Elizabeth Rowley, DrPH[2]; Melissa S. Stockwell, MD, MPH[3]; Sara Y. Tartof, PhD, MPH[4,5]; Kristin Dascomb, MD, PhD[6]; Stephanie A. Irving, MHS[7]; Brian Dixon, PhD, MPA[8,9]; Sarah W. Ball, ScD[2]; Mark W. Tenforde, MD, PhD[1]; Gabriela Vazquez-Benitez, PhD, MSc[10]; Ashley B. Stephens, MD[3]; Jungmi Han, BS[3]; Karthik Natarajan, PhD[3]; S. Bianca Salas, MPH[4]; Cassandra Bezi, MPH[4]; Lina S. Sy, MPH[4]; Bruno Lewin, MD[4]; Tamara Sheffield, MD, MPA, MPH[6]; Julie Arndorfer, MPH[6]; Daniel Bride, MS[6]; Josh Van Otterloo, MS[6]; Allison L. Naleway, PhD[7]; Padma D. Koppolu, MPH[7]; Shaun Grannis, MD, MS[8,11]; William Fadel, PhD[8,9]; Colin Rogerson, MD, MPH[8,11]; Tom Duszynski, PhD[9]; Sarah E. Reese, PhD[2]; Patrick K. Mitchell, ScD[2]; Sean Chickery, DHSc[2]; Heidi L. Moline, MD MPH[1,13]; Morgan Najdowski, MPH[1,12]; Allison Avrich Ciesla, PhD[1,12]; Emily L. Reeves, MPH[1]; Malini DeSilva, MD, MPH[10]; Katherine E. Fleming-Dutra, MD[1]; Ruth Link-Gelles, PhD, MPH[1,13]

Affiliations:

[1]National Center for Immunization and Respiratory Diseases, Centers for Disease Control and Prevention, Atlanta, GA, USA
[2]Westat, Rockville, MD, USA
[3]Columbia University Irving Medical Center, New York, NY, USA
[4]Kaiser Permanente Southern California, Pasadena, CA, USA
[5]Kaiser Permanente Bernard J Tyson School of Medicine, Pasadena, CA, USA
[6]Intermountain Health, Salt Lake City, UT, USA
[7]Kaiser Permanente Center for Health Research, Portland, OR, USA
[8]Center for Biomedical Informatics, Regenstrief Institute, Indianapolis, IN, USA
[9]Richard M. Fairbanks School of Public Health, Indiana University, Indianapolis, IN, USA
[10]HealthPartners Institute, Minneapolis, MN, USA
[11]Indiana University School of Medicine, Indianapolis, IN, USA
[12]Eagle Health Analytics, San Antonio, TX, USA
[13]United States Public Health Service Commissioned Corps, Rockville, MD, USA

Contents

Supplementary Table 1: Acute illness categories and corresponding International Classification of Diseases, 10th Revision (ICD-10) discharge codes 3

Supplementary Table 2: Description of site-specific methods used to link maternal and infant records in the Virtual SARS-CoV-2, Influenza, and Other respiratory viruses Network (VISION), October 2023 – March 2024 7

Supplementary Table 3: Underlying medical condition categories and corresponding International Classification of Diseases, 10th Revision (ICD-10) discharge codes 8

Supplementary Methods 1: Description of methods used to ascertain additional variables considered in sensitivity analyses. 11

Supplementary Methods 2: Description of methods used to estimate nirsevimab effectiveness among infants in their first RSV season using a model that adjusted for propensity-to-be-immunized. 11

Supplementary Table 4: Explanatory variables used to estimate the propensity to receive nirsevimab immunization. 13

Supplementary Table 5: Description of sensitivity and secondary analyses. 16

Supplementary Figure 1: Analytic population flow for assessment of first season nirsevimab product effectiveness against RSV-associated ED encounters – VISION, October 8, 2023 – March 31, 2024 20

Supplementary Table 6. Nirsevimab effectiveness against RSV-associated ED encounters among infants born preterm or with underlying medical conditions, VISION Network, Oct 8, 2023-March 31, 2024 21

Supplementary Table 7: Sensitivity and secondary analyses results for estimation of nirsevimab product effectiveness against RSV-associated ED encounters among infants in their first RSV season. 22

Supplementary Table 8: Evaluating the effect of including additional covariates in logistic regression models for estimation of nirsevimab product effectiveness against RSV-associated ED encounters among infants in their first RSV season. 25

Supplementary Figure 2: Analytic population flow for assessment of first season nirsevimab product effectiveness against RSV-associated hospitalization – VISION, October 8, 2023 – March 31, 2024 27

Supplementary Table 9. Nirsevimab effectiveness against RSV-associated hospitalization among infants born preterm or with underlying medical conditions, VISION Network, Oct 8, 2023-March 31, 2024 28

Supplementary Table 10: Sensitivity and secondary analyses results for estimation of nirsevimab product effectiveness against RSV-associated hospitalization among infants in their first RSV season. 29

Supplementary Table 11: Evaluating the effect of including additional covariates in logistic regression models for estimation of nirsevimab product effectiveness against RSV-associated hospitalization among infants in their first RSV season. 31

Supplementary Table 1: Acute illness categories and corresponding International Classification of Diseases, 10th Revision (ICD-10) discharge codes

Disease Condition	ICD-10 codes	Included in ARI definition	Included in RLI definition
**COVID-19 Pneumonia**			
Pneumonia due to SARS-associated coronavirus	J12.81	X	X
Pneumonia due to coronavirus disease 2019	J12.82	X	X
**Influenza Pneumonia**			
Influenza due to identified novel influenza A virus with pneumonia	J09.X1	X	X
Influenza due to other identified influenza virus with pneumonia	J10.0*	X	X
Influenza due to other identified influenza virus with unspecified type of pneumonia	J10.00	X	X
Influenza due to other identified influenza virus with the same other identified influenza virus pneumonia	J10.01	X	X
Influenza due to other identified influenza virus with other specified pneumonia	J10.08	X	X
Influenza due to unidentified influenza virus with pneumonia	J11.0*	X	X
Influenza due to unidentified influenza virus with unspecified type of pneumonia	J11.00	X	X
Influenza due to unidentified influenza virus with specified pneumonia	J11.08	X	X
**Other Viral Pneumonia**	J12.0, J12.1, J12.2, J12.3, J12.89, J12.9	X	X
**Bacterial and Other Pneumonia**			
Streptococcus pneumoniae pneumonia	J13	X	X
Hemophilus influenzae pneumonia	J14	X	X
Other bacterial pneumonia	J15*	X	X
Pneumonia due to other specified organism	J16*	X	X

Pneumonia in infectious diseases classified elsewhere	J17	X	X
Pneumonia, unspecified organism	J18*	X	X
**Influenza Disease**	J09.X2, J09.X3, J09.X9, J10.1, J10.2, J10.8*, J11.1, J11.2, J11.8*	X	X
**Acute respiratory distress syndrome**	J80	X	X
**Asthma acute exacerbation**	J45.21, J45.22, J45.31, J45.32, J45.41, J45.42, J45.51, J45.52, J45.901, J45.902	X	X
**Respiratory failure**			
Acute respiratory failure	J96.0*	X	X
Acute and chronic respiratory failure	J96.2*	X	X
Respiratory arrest	R09.2	X	X
Respiratory failure, unspecified	J96.9*		X
**Other acute lower respiratory tract infections**			
Acute bronchitis	J20*	X	X
Acute bronchiolitis	J21*	X	X
Unspecified acute lower respiratory infection	J22	X	X
Bronchitis, not specified as acute or chronic	J40	X	X
Simple and mucopurulent chronic bronchitis	J41*	X	X
Unspecified chronic bronchitis	J42	X	X
Emphysema	J43*	X	X
Bronchiectasis	J47*	X	X
Abscess of lung and mediastinum	J85*	X	X
Gangrene and necrosis of lung	J85.0	X	X
Abscess of lung without pneumonia	J85.2	X	X
Abscess of mediastinum	J85.3	X	X
Abscess of lung with pneumonia	J85.1	X	X
Pyothorax	J86*	X	X

Respiratory syncytial virus as the cause of diseases classified elsewhere	B97.4	X	X
**Acute sinusitis**	J01*	X	X
**Acute upper respiratory tract infections**	J00*, J02*, J03*, J04*, J05*, J06*	X	X
**Viral illness, not otherwise specified**	B34.9		X
**Acute Respiratory Illness Signs and Symptoms**			
**Hemoptysis**	R04.2	X	X
**Cough**	R05, R05.1, R05.2, R05.4, R05.8, R05.9	X	X
**Dyspnea unspecified**	R06.00	X	X
Shortness of breath	R06.02	X	X
Acute respiratory distress	R06.03	X	X
Stridor	R06.1	X	X
Wheezing	R06.2	X	X
Other abnormalities of breathing	R06.8	X	X
Apnea, not elsewhere classified	R06.81	X	X
Tachypnea, NEC	R06.82	X	X
Other abnormalities of breathing/ Other symptoms involving head & neck	R06.89	X	X
**Chest pain on breathing/ painful respiration**	R07.1	X	X
**Asphyxia and hypoxemia**	R09.0*	X	X
Asphyxia	R09.01	X	X
Hypoxemia	R09.02	X	X
**Pleurisy**	R09.1	X	X
**Respiratory arrest**	R09.2	X	X
**Abnormal sputum**	R09.3	X	X
**Other specified symptoms and signs involving the circulatory and respiratory systems**	R09.8*	X	X
**Acute Non-respiratory Illness Signs and Symptoms**			

**Sepsis - Symptoms and signs specifically associated with systemic inflammation and infection**	R65*		X
**Shock, unspecified**	R57.9		X
**Irritable/fussy infant**	R68.12		X
**Respiratory illnesses originating in perinatal period**			
Respiratory distress of newborn	P22*		X
Congenital pneumonia	P23*		X
Interstitial emphysema and related conditions	P25*		X
Other respiratory conditions originating in perinatal period	P28*		X
Congenital viral diseases	P35*		X
Bacterial sepsis of newborn	P36*		X
Other infections specific to perinatal period	P39*		X

ARI: acute respiratory illness | RLI: RSV-like illness

*Includes all sub-codes

Supplementary Table 2: Description of site-specific methods used to link maternal and infant records in the Virtual SARS-CoV-2, Influenza, and Other respiratory viruses Network (VISION), October 2023 – March 2024

Site	Linkage method
**A**	A maternal-infant linkage file is populated using data recorded in a delivery registry table using hospital registries, medical records, and health plan administrative data for the monthly update and the birth certificate data for the annual update.
**B**	For infants born within the hospital system, a direct data linkage is created using data within the electronic health record. For infants born outside the health system, indirect linkage may be determined using data fields for insurance guarantor and/or emergency contact to identify maternal contact information recorded in infant encounter records.
**C**	For infants born within the hospital system, direct linkage of maternal and infant records is available within the EHR. When direct linkage is not available, maternal and infant records are linked based on an algorithm. The algorithm imputes linkages based on data from membership, health plan coverage, common latitude/longitude coordinates, and birth and obstetric records.
**D**	For infants born within the hospital system (either internal or contract hospital), direct linkage of maternal and infant records is available via hospital delivery records. Direct linkage is also available based on hospital account or patient records. When direct linkage is not available, an algorithmic linkage is determined. For algorithmic linkage two criteria must be satisfied: (1) the subscriber group is the same in both the maternal and infant records OR address is the same in the maternal and infant records AND (2) additional data are available that suggest a biological linkage, including maternal record of live birth close to the infant date of birth.
**E**	Organization has access to state natality data (birth certificates). The data analyst will first use an existing maternal child linkage indicator. Otherwise, the analyst will implement the MPRINT maternal child linkage algorithm. The MPRINT maternal child linkage algorithm matches mothers to their children using demographic data from electronic health records (EHRs). The algorithm is a multi-step process that involves, in order, business rules, blocking scheme, and a prediction model.
**F**	A maternal-infant linkage is determined by using the delivery record from EHR to identify the mother and infant.<br><br>Matches in the patient relationship table compare the mother's name, insurance payor ID, home phone number, email address, or cell phone number.

Supplementary Table 3: Underlying medical condition categories and corresponding International Classification of Diseases, 10th Revision (ICD-10) discharge codes

Disease Condition	ICD-10 Codes
**Respiratory Disease:**	
Asthma	J45.*
Other chronic lung disease	D86.0, E88.01, J47.*, J66.*, J67.0, J67.1, J67.2, J67.3, J67.4, J67.5, J67.6, J67.7, J67.8, J68.*, J70.*,J81.1, J84.*, J95*, J96.1*, J99.*, P26.*, P27.*, B39.*, B40.0, B40.2, B41.0, B44.0, B44.1, B45.*, B46.0, A15.*, A31.0
Apnea	G47.3*
Chronic lung disease of prematurity	P27.0, P27.1, P27.8, P27.9
Cystic fibrosis	E84*
Congenital airway abnormality	Q34*, Q30*, Q31*, Q32*, Q39*, Q79.0
Reactive airway disease	J45.901, J45.902, J45.909
**Cardiovascular Disease:**	
Heart failure	I50.*
Ischemic heart disease	I21.*, I22.*, I23.*, I24.*, I25.*
Hypertension	I10.*, I11.*, I13.*, I15.*
Other heart disease	I01.*, I02.0, I09.*, I27.*, I28.*, I31.*, I42.*, I43.*, I44.*, I46.*, I51.0, I51.1, I51.2, I51.3, I51.5, I51.7, I51.8*, I51.9, I52.*, I97.0, I97.1*, M31.0, M31.1*, M31.2, M31.4, M31.6, M31.7, M31.8, M31.9, Z95.*, Z98.61, I71.*, I72.*, I73.*, I74.*, I75.*, I79.*
Pulmonary embolism	I26.*
Heart valve disorders	I05.*, I06.*, I07.*, I08.*, I34.*, I35.*, I36.*, I37.*
Atrial fibrillation and flutter	I48.*
Congenital heart disease	I50.9, I42.9, Q21*, Q22*, Q23*, Q24*, Q25*, Q26*, Q27.0, Q27.3*, Q27.4, Q27.8, Q27.9, Q28*, Q33*, P29.30
**Cerebrovascular Disease:**	
Stroke	I60.*, I61.*, I63.*
Other cerebrovascular disease	I62.*, I68.*, I69.*
**Neurological and Musculoskeletal Disease:**	

Neurological/musculoskeletal disorder	H49.81*, M12.0*, M36.0, E75.02, E75.19, E75.4, F71.*, F72.*, F73.*, F84.2, G10.*, G11.*, G12.*, G13.*, G14.*, G20.*, G21.*, G23.*, G24.*, G25.*, G26.*, G31.*, G32.*, G35.*, G36.*, G37.*, G45.*, G46.*, G60.*, G61.*, G62.*, G63.*, G64.*, G70.*, G73.*, G81.*, G82.*, G83.*, G90.3, G91.*, G93.*, G94.*, G95.*, G99.2, P91.*, Q00.*, Q01.*, Q02.*, Q03.*, Q04.*, Q05.*, Q06.*, Q07.*, Q76.*, Q77.*, Q78.*, Q79.1*, Q79.2*,Q79.3*,Q79.4*,Q79.5*,Q79.6*,Q79.8*, Q79.9*,Q85.*, Q87.4*, Q91.*, Q92.*, Q93.*, Q96.*, R41.*, R53.2, R54.*
Down Syndrome	Q90.*
Muscular dystrophy	G71.0*
Neurological conditions	G40.*, R56.*, P90, P91.0, P52.*, G80.*, E70*, E71*, E72*, E74*, E75.2*, E76*, E77*, E78*, E79*, E80*, P70*, P71*, P72*, P74*, P94*
**Hematologic Disease:**	
Blood disorder	D55.*, D56.8, D58.*, D59.*, D60.*, D61.*, D64.0, D64.1, D64.2, D64.3, D64.4, D64.8*, D65.*, D66.*, D67.*, D68.*
Sickle cell disease	D56.0, D56.1, D56.2, D56.4, D56.5, D56.9, D57.0*, D57.1, D57.2*, D57.4*, D57.8*
**Endocrine/Metabolic Disease:**	
Diabetes type I	E10.*
Diabetes type II	E11.*
Diabetes due to underlying condition or other specified diabetes	E08.*, E09.* E13.*
Other metabolic disease	E00.*, E01.*, E03.*, E05.*, E06.*, E15.* , E16.*, E20.*, E21.*, E22.*, E23.*, E24.*, E25.*, E26.*, E27.*, E28.*, E29.*, E31.*, E32.*, E34.*, E83.*, E85.*, E88.02, E88.09, E88.1, E88.2, E88.3, E88.4*, E88.8*, E88.9.
**Renal Disease:**	
Any renal underlying medical condition	I12.*, I13.* N01.*, N02.*, N03.*, N04.*, N05.*, N06.*, N07.*, N08.*, N11.*, N14.*, N15.*, N16.*, N18.*, N25.*, N26.*, N28.*, Q27.1, Q27.2, Q60.*, Z49.*, Z91.15, Z94.0, Z99.2
**Gastrointestinal and Hepatic Disease:**	
Liver disease	B18.*, I81.*, I85.*, K70.*, K71.*, K72.*, K73.*, K74.*, K75.*, K76.*, K77.*
Chronic gastrointestinal condition	K50*, K51*, K52*
**Clinical Obesity:**	

Clinical obesity	E66.*, Z68.3, Z68.4
**Clinical Underweight:**	
Clinically underweight	R63.6, F50.0*, E40.*, E41.*, E42.*, E43.*, E44.*, E45.*, E46.*
**Other conditions**	
Premature birth	P07.*
Developmental delay	R62.*
Technology dependence	Z93.0, Z93.1, Z99.0, Z99.11, Z99.81, Z99.89

*Includes all sub-codes

Supplementary Methods 1: Description of methods used to ascertain additional variables considered in sensitivity analyses.

Additional variables considered in subgroup and sensitivity analyses were collected from the EHR, including preterm birth status, gestational age at birth, birthweight, and neonatal intensive care unit (NICU) admission at birth, receipt of mechanical ventilation at birth. Children were considered born preterm if an International Classification of Disease, 10th revision (ICD-10) code corresponding to preterm birth (P07.*) was listed as a discharge diagnosis at the time of the encounter or the gestational age at birth recorded in the EHR was <37 weeks. Gestational age at birth was categorized as early preterm (<34 weeks), late preterm (34-36 weeks), early term (37-38 weeks), full term (39-40 weeks), late term (41 weeks), and post term (≥42 weeks). Birthweight was categorized as ≤1,200 g, 1201-1499 g, 1500-2499 g, ≥2,500 g. For each of these variables, missing was considered a separate category.

Supplementary Methods 2: Description of methods used to estimate nirsevimab effectiveness among infants in their first RSV season using a model that adjusted for propensity-to-be-immunized.

In a secondary analysis, nirsevimab effectiveness was estimated with the use of weighted multivariable logistic-regression models adjusted for age, race and ethnicity, sex, calendar day (days since October 1, 2023), geographic region, and inverse propensity-to-be-immunized weights. Using established methods for estimating propensity scores within case–control studies,[1] we first estimated propensity-to-be-immunized scores among test-negative control encounters and then used the fitted model to calculate propensity-to-be-immunized scores for test-positive encounters.

Each observation was weighted by the inverse of the propensity to be immunized, with generalized boosted regression trees used to estimate the propensity to be immunized with a set of explanatory variables (Supplementary Table 4).[2] Weights were truncated at the 99.9 percentile of the distribution of weights.[3] Separate weights were calculated for the model estimating nirsevimab effectiveness against

RSV-associated emergency department encounters and the model estimating nirsevimab effectiveness against RSV-associated hospitalization. Age, race and ethnicity, sex, calendar day (days since October 1, 2023), and geographic region were included both in weight calculations and as covariates in the nirsevimab effectiveness regression models.[4]

[1]Månsson R, Joffe MM, Sun W, Hennessy S. On the estimation and use of propensity scores in case-control and case-cohort studies. *Am J Epidemiol* 2007;166:332-339.

[2]McCaffrey DF, Ridgeway G, Morral AR. Propensity score estimation with boosted regression for evaluating causal effects in observational studies. *Psychol Methods* 2004;9:403-425.

[3]Austin PC, Stuart EA. Moving towards best practice when using inverse probability of treatment weighting (IPTW) using the propensity score to estimate causal treatment effects in observational studies. *Stat Med* 2015;34:3661-3679.

[4]Nguyen TL, Collins GS, Spence J, et al. Double-adjustment in propensity score matching analysis: choosing a threshold for considering residual imbalance. *BMC Med Res Methodol* 2017;17:78-78.

Supplementary Table 4: Explanatory variables used to estimate the propensity to receive nirsevimab immunization.

Variable	Considered for model estimating nirsevimab effectiveness against RSV-associated ED encounters	Considered for model estimating nirsevimab effectiveness against RSV-associated hospitalization
**Event Characteristics**		
Number of additional encounters combined under encounter ID	X*	X*
Number of days since October 8, 2023, at date of encounter	X*	X*
**Setting Characteristics**		
Number of hospital beds		X
Hospital ownership		X
Hospital type		X
Urban-rural classification of facility	X	X
Geographic region	X	X
**Patient Characteristics**		
Age	X*	X*
Date of birth	X	X
Estimated or known date of birth	X	X
Sex	X	X*
Race	X	X
Hispanic/LatinX ethnicity	X*	X
Medicaid status	X*[†]	X
**Underlying Conditions**		
Gestational age at birth	X	X
Birthweight	X	X*[‡]
NICU admission at birth	X	X*[§]
Length of stay during NICU admission at birth	X*[¶]	X[§]

ECMO at birth	X	X
IMV at birth	X	X
Other respiratory support at birth	X	X*,**
Asthma	X	X
Other chronic lung disease	X	X*
Apnea	X	X
Congenital heart disease	X	X*
Neurological/musculoskeletal disorder	X	X*
Down Syndrome	X	X
Muscular dystrophy	X	X
Neurological conditions	X	X*
Sickle cell disease	X	X
Endocrine/Metabolic Disease	X	X*
Renal Disease	X	X
Chronic gastrointestinal condition	X	X
Clinical obesity	X	X
Clinically underweight	X	X
Preterm birth	X	X*
Developmental delay	X	X
Technology dependence	X	X
Chronic lung disease of prematurity	X	X
Cystic fibrosis	X	X
Congenital airway abnormalities	X	X
Reactive airway disease	X	X
**Maternal/infant record linkage**		
Linkage to maternal RSV vaccination records	X	X*
Type of linkage to maternal RSV vaccination records[††]	X	X*
**Other Vaccination History**		
Infant COVID-19 vaccination status	X	X
Infant influenza vaccination status	X	X

Maternal COVID-19 vaccination during pregnancy	X	X
Maternal influenza vaccination during pregnancy	X	X
**Number of well child visits prior to encounter**		
Number of well child visits prior to encounter	X*,[‡‡]	X

X = Variable considered for inclusion | NICU = Neonatal intensive care unit | ECMO = extracorporeal membrane oxygenation | IMV = invasive mechanical ventilation

*Indicates variable included in final inverse propensity-to-be-vaccinated weights.
[†]Medicaid status unknown for 361 (7%) of ED encounters.
[‡]Birthweight unknown for 386 (38%) of hospitalizations.
[§]NICU admission at birth unknown for 487 (48%) of hospitalizations.
[¶]NICU admission at birth unknown for 2,595 (52%) of ED encounters.
**Need for other respiratory support at birth unknown for 484 (47%) of hospitalizations.
[††]Indicates direct or indirect linkage of maternal and infant records.
[‡‡]Indicates the number of well child visits recorded in an infant's electronic health record (EHR) prior to the ED encounter. Distribution of number of well-child visits prior to the ED encounter: 0 (4,257 [85%]), 1-3 (456 [9%]), 4-7 (326 [6%]).

Supplementary Table 5: Description of sensitivity and secondary analyses.

Sensitivity Analysis	Description
Varying date of eligible encounters based on RSV circulation and/or nirsevimab implementation	
Analytic period: October 8, 2023, through region-specific offset, determined based on NREVSS surveillance data*	Because RSV circulation can vary by geographic region and over-capture of RSV-negative, vaccinated encounters during time periods of extremely low RSV circulation at sites could bias VE, we varied the dates of eligible encounters based on 2023–2024 RSV season end dates by HHS region derived from data collected by the NREVSS.†
Analytic period: October 8, 2023, through site-specific offset, determined based on VISION data‡	Because RSV circulation can vary by geographic region and over-capture of RSV-negative, vaccinated encounters during time periods of extremely low RSV circulation at sites could bias VE, we varied the dates of eligible encounters based on the last observed encounter for RLI with a positive RSV test at each site.
Analytic period: Region-specific onset determined based on NREVSS surveillance data* through March 31, 2024	Because RSV circulation can vary by geographic region and over-capture of RSV-negative, vaccinated encounters during time periods of extremely low RSV circulation at sites could bias VE, we varied the dates of eligible encounters based on 2023–2024 RSV season start dates by HHS region derived from data collected by the NREVSS.†
Analytic period: region-specific onset and offset, determined based on NREVSS surveillance data*	Because RSV circulation can vary by geographic region and over-capture of RSV-negative, vaccinated encounters during time periods of extremely low RSV circulation at sites could bias VE, we varied the dates of eligible encounters based on 2023–2024 RSV season start and end dates by HHS region derived from data collected by the NREVSS.†
Analytic period: region-specific onset determined based on NREVSS surveillance data* through site-specific offset determined based on VISION data‡	Because RSV circulation can vary by geographic region and over-capture of RSV-negative, vaccinated encounters during time periods of extremely low RSV circulation at sites could bias VE, we varied the dates of eligible encounters based on 2023–2024 RSV season start dates by HHS region derived from data collected by the NREVSS † and the last observed encounter for RLI with a positive RSV test at each site.
Analytic period: site-specific onset based on date of nirsevimab implementation§ through March 31, 2024	Because nirsevimab may have been implemented at different times at each site and over-capture of unimmunized, RSV-positive encounters due to low uptake of nirsevimab could bias VE, we varied the dates of eligible encounters based on the date nirsevimab was implemented at each site.
Analytic period: site-specific onset based on date of nirsevimab implementation§ through region-specific offset based on NREVSS surveillance data*	Because nirsevimab may have been implemented at different times at each site and over-capture of unimmunized, RSV-positive encounters due to low uptake of nirsevimab could bias VE, we varied the dates of eligible encounters based on the date nirsevimab was implemented at each site and 2023–

	2024 RSV season end dates by HHS region derived from data collected by the NREVSS.†
Restricting to molecular-positive case encounters	
Excluding RSV-antigen positive cases	Test sensitivity and specificity can influence VE estimates, with specificity having the largest impact on estimates.¶ In a meta-analysis comparing RSV antigen tests to molecular tests, the specificity of antigen tests was over 95%,** indicating inclusion of encounters positive by RSV antigen tests was reasonable. Nevertheless, to examine the potential biasing effect of including RSV antigen-positive encounters due to potential differences in assay sensitivity and specificity between antigen and molecular tests, we excluded these encounters from the analysis.
Excluding controls testing positive for SARS-CoV-2 or influenza	
Excluding SARS-CoV-2 and flu-positive controls	To examine the potential biasing effect of including RSV-negative (i.e., control) encounters that were positive for SARS-CoV-2 or influenza due to the possible correlation of vaccination behaviors, we excluded these encounters from the analysis.††
Including infants with evidence of nirsevimab receipt 0-6 days prior to the encounter	
Including infants with evidence of nirsevimab receipt 0-6 days prior to the encounter	Children whose RLI encounter was within 0-6 days after nirsevimab receipt could have been incubating/infected with RSV prior to nirsevimab receipt. Additionally, in clinical trials, peak neutralizing antibody concentration levels were reached by day 6 after administration.‡‡ Thus, our main analysis excluded infants with evidence of nirsevimab receipt 0-6 days prior to the encounter. To examine the possible effect of excluding infants with evidence of nirsevimab receipt 0-6 days prior to the encounter, we included these encounters in the analysis.
Excluding infants with immunocompromising conditions	
Excluding infants with immunocompromising conditions	Because nirsevimab is a passive immunization, no immune response is required by the recipient for protection to be conferred; thus, infants with immunocompromising conditions were included in the analysis. To examine the possible effect of including infants with immunocompromise, we excluded encounters among these infants from the analysis.
Restricting encounters to those with ARI	
Limiting to ARI encounters	To examine the possible effect of including discharge diagnoses consistent with RLI but broader than ARI, encounters were limited to those with at least one ICD-10 discharge diagnosis code consistent with ARI.
Excluding site testing a relatively low proportion of encounters	
Excluding 1 site with relatively low RSV testing	To examine the effect of including sites that tested a comparatively lower proportion of RLI encounters, we

	excluded the site with the lowest proportion of tested encounters from the analysis.
Secondary analysis using a weighted multivariable logistic regression model	
Additional adjustment for inverse propensity-to-be-immunized weights	We estimated the adjusted odds ratio using multivariable logistic regression models adjusted for age, race and ethnicity, sex, calendar day (days since October 1, 2023), HHS geographic region, and inclusion of weights based on the inverse of the propensity-for-immunization scores (Supplementary Methods 2 and Supplementary Table 4).
Varying time since dose categories	
Varying the grouping of times since dose	In our main analysis we estimate effectiveness by time since dose, grouping 7-59 days and ≥60 days.[§§] As the mean half-life of nirsevimab in clinical trials was estimated to be 59 days, with nirsevimab levels being higher than the targeted effective concentration threshold up to 151 days post dose,[¶¶] we evaluated whether evidence of waning may be more apparent using different groupings (i.e., 7-89 days and ≥90 days).

ARI: acute respiratory illness | RSV: respiratory syncytial virus | RLI: RSV like illness | NREVSS: National Respiratory and Enteric Virus Surveillance System

*Start and end dates based on the first and last of 2 consecutive weeks when the percentage of PCR tests positive for RSV was ≥3%. HHS Region 2 (Columbia University Irving Medical Center) Start: October 28, 2023, End: January 27, 2024 | HHS Region 5 (HealthPartners and Regenstrief Institute) Start: October 21, 2023, End: March 9, 2024 | HHS Region 8 (Intermountain Health) Start: November 18, 2023, End: April 6, 2024 | HHS Region 9 (Kaiser Permanente Southern California) Start: September 30, 2023 [while this is the onset of the RSV season, eligible encounters occurred on or after October 8, 2023, to coincide with nirsevimab availability in the United States], End: February 24, 2024 | HHS Region 10 (Kaiser Permanente Northwest) Start: October 28, 2023, End: March 2, 2024

[†]https://www.cdc.gov/surveillance/nrevss/index.html

[‡]End dates based on the last observed encounter for RSV-like illness with a positive RSV test. Columbia University Irving Medical Center April 14, 2024 | HealthPartners April 14, 2024 | Regenstrief Institute April 8, 2024 | Intermountain Health April 28, 2024 | Kaiser Permanente Southern California May 4, 2024 | Kaiser Permanente Northwest April 11, 2024

[§]Start dates based on the date nirsevimab was implemented, determined based on the date on or after August 3, 2023, that was 7 days after the first instance of nirsevimab receipt recorded in the data, regardless of whether it occurred in a child with an encounter for RSV-like illness. Columbia University Irving Medical Center October 19, 2023 | HealthPartners October 16, 2023 | Regenstrief Institute October 8, 2023 | Intermountain Health October 9, 2023 | Kaiser Permanente Southern California October 22, 2023 (date reported by site) | Kaiser Permanente Northwest October 20, 2023

[¶]Jackson ML and Rothman KJ. Effects of imperfect test sensitivity and specificity on observational studies of influenza vaccine effectiveness. Vaccine. 2015;33(11):1313-1316.

** Onwuchekwa C, Moreo LM, Menon S, et al. Underascertainment of Respiratory Syncytial Virus Infection in Adults Due to Diagnostic Testing Limitations: A Systematic Literature Review and Meta-analysis. *J Infect Dis*. 2023;228(2):173-184.

[††]Doll MK, Pettigrew SM, Ma J, Verma A. Effects of Confounding Bias in Coronavirus Disease 2019 (COVID-19) and Influenza Vaccine Effectiveness Test-Negative Designs Due to Correlated Influenza and COVID-19 Vaccination Behaviors. *Clin Infect Dis*. 2022;75(1):e564-e571

[‡‡]https://www.accessdata.fda.gov/drugsatfda_docs/label/2023/761328s000lbl.pdf

[§§]Nirsevimab effectiveness by time since dose was only estimated against emergency department encounters due to sample size limitations.

[¶¶]Griffin MP, Yuan Y, Takas T, et al. Single-Dose Nirsevimab for Prevention of RSV in Preterm Infants. *N Engl J Med* 2020; **383**(5): 415-25.

Supplementary Figure 1: Analytic population flow for assessment of first season nirsevimab product effectiveness against RSV-associated ED encounters – VISION, October 8, 2023 – March 31, 2024

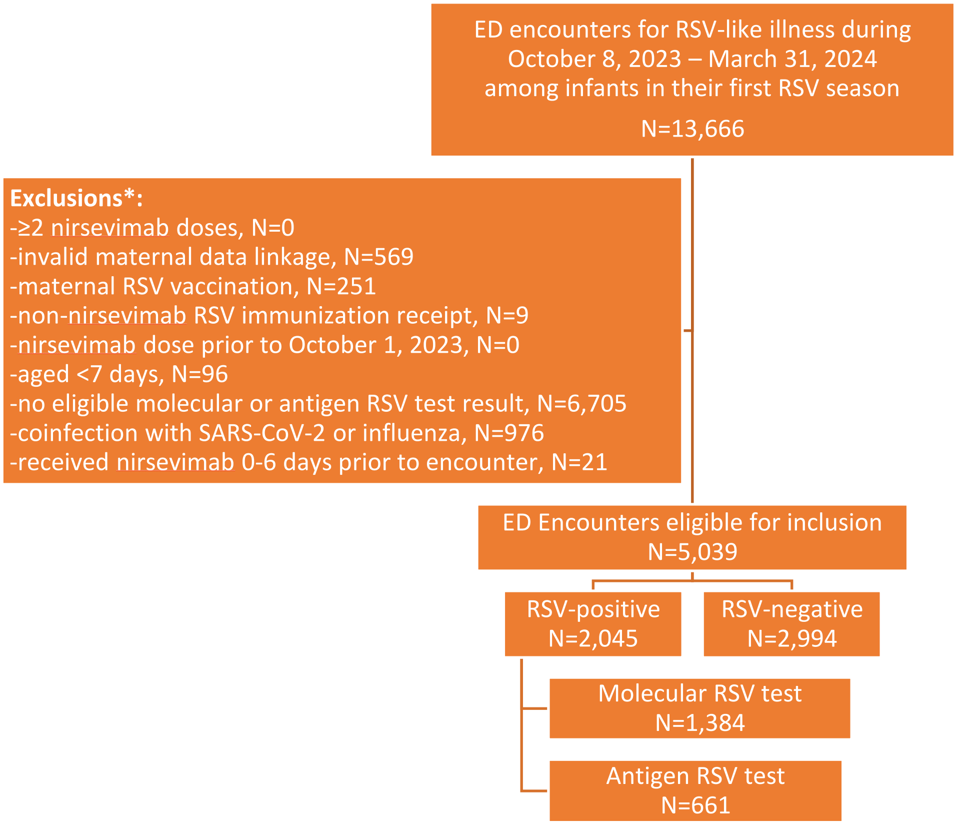

*Exclusions applied in order listed. Some encounters might have met ≥1 exclusion criteria.

Supplementary Table 6. Nirsevimab effectiveness against RSV-associated ED encounters among infants born preterm or with underlying medical conditions, VISION Network, Oct 8, 2023-March 31, 2024

Category | nirsevimab dosage pattern	Total ED encounters*	RSV-positive ED encounters N (Row %)	Median days since dose (IQR)	Adjusted PE (95% CI)†
Infants born preterm				
No nirsevimab doses	359	132 (37)	N/A	Ref
Nirsevimab, 7-164 days prior	70	6 (9)	46 (20-81)	86 (58-95)
Infants with ≥1 UMC				
No nirsevimab doses	149	52 (35)	N/A	
Nirsevimab, 7-164 days prior	29	2 (7)	48 (27-78)	89 (15-98)

RSV = respiratory syncytial virus; ED = emergency department; IQR = interquartile range; CI = confidence interval; Ref = reference group; PE = product effectiveness; VISION = Virtual SARS-CoV-2, Influenza, and Other respiratory viruses Network; UMC = underlying medical condition

*Encounters included those among infants in their first RSV season with a diagnosis of RSV-like illness (RLI), excluding infants with evidence of maternal RSV vaccination and infants who received nirsevimab <7 days prior to the index date for the encounter. RLI was defined as ≥1 International Classification of Disease 10th Revision discharge diagnosis code corresponding to one or more of the following: COVID-19 pneumonia, influenza pneumonia, other viral pneumonia, influenza disease, bacterial pneumonia, acute respiratory distress syndrome, asthma exacerbation, respiratory failure, other acute lower respiratory tract infection, sinusitis, acute upper respiratory tract infections, acute respiratory illness signs and symptoms, viral illness not otherwise specified, sepsis, respiratory failure, irritable/fussy infant, respiratory distress of newborn, congenital pneumonia, interstitial emphysema and related conditions, other respiratory conditions originating in the perinatal period, congenital viral diseases, bacterial sepsis of newborn, or other infections specific to the perinatal period.

†PE was calculated as (1 – adjusted odds ratio) x 100%, with adjusted odds ratio calculated using logistic regression, adjusting for age, race and ethnicity, sex, calendar day, and geographic region.

Supplementary Table 7: Sensitivity and secondary analyses results for estimation of nirsevimab product effectiveness against RSV-associated ED encounters among infants in their first RSV season.

Sensitivity/Secondary Analysis	Adjusted PE (95% CI)¶
Main Analysis	77 (69-83)
Varying date of eligible encounters based on RSV circulation and/or nirsevimab implementation	
Analytic period: October 8, 2023, through region-specific offset, determined based on NREVSS surveillance data*†	76 (67-82)
Analytic period: October 8, 2023, through site-specific offset, determined based on VISION data‡	78 (71-84)
Analytic period: Region-specific onset determined based on NREVSS surveillance data*† through March 31, 2024	77 (68-83)
Analytic period: region-specific onset and offset, determined based on NREVSS surveillance data*†	75 (66-82)
Analytic period: region-specific onset determined based on NREVSS surveillance data*† through site-specific offset determined based on VISION data‡	78 (70-84)
Analytic period: site-specific onset based on date of nirsevimab implementation§ through March 31, 2024	77 (69-83)
Analytic period: site-specific onset based on date of nirsevimab implementation§ through region-specific offset based on NREVSS surveillance data*†	76 (66-82)
Restricting to molecular-positive case encounters	
Excluding RSV-antigen positive cases	78 (69-85)
Excluding controls testing positive for SARS-CoV-2 or influenza	
Excluding SARS-CoV-2 and flu-positive controls	77 (69-83)
Including infants with evidence of nirsevimab receipt 0-6 days prior to the encounter	
Including infants with evidence of nirsevimab receipt 0-6 days prior to the encounter	76 (67-82)
Excluding infants with immunocompromising conditions	
Excluding infants with immunocompromising conditions	77 (69-83)
Restricting encounters to those with ARI	
Limiting to ARI encounters	77 (69-83)
Excluding site testing a relatively low proportion of encounters	
Excluding 1 site with relatively low RSV testing	77 (68-84)
Secondary analysis using a weighted multivariable logistic regression model	
Additional adjustment for inverse propensity-to-be-immunized weights	76 (66-83)**
Varying time since dose categories	
Varying the grouping of times since dose††	7-89 days prior: 76 (67-82)<br>90-164 days prior: 86 (59-95)

RSV: respiratory syncytial virus | CI: confidence interval | ARI: acute respiratory illness | PE: product effectiveness | NREVSS: National Respiratory and Enteric Virus Surveillance System

*Start and end dates based on the first and last of 2 consecutive weeks when the percentage of PCR tests positive for RSV was ≥3%. HHS Region 2 (Columbia University Irving Medical Center) Start: October 28, 2023, End: January 27, 2024 | HHS Region 5 (HealthPartners and Regenstrief Institute) Start: October 21, 2023, End: March 9, 2024 | HHS Region 8 (Intermountain Health) Start: November 18, 2023, End: April 6, 2024 | HHS Region 9 (Kaiser Permanente Southern California) Start: September 30, 2023 [while this is the onset of the RSV season, eligible encounters occurred on or after October 8, 2023, to coincide with nirsevimab availability in the United States], End: February 24, 2024 | HHS Region 10 (Kaiser Permanente Northwest) Start: October 28, 2023, End: March 2, 2024

†https://www.cdc.gov/surveillance/nrevss/index.html

‡End dates based on the last observed encounter for RSV-like illness with a positive RSV test. Columbia University Irving Medical Center April 14, 2024 | HealthPartners April 14, 2024 | Regenstrief Institute April 8, 2024 | Intermountain Health April 28, 2024 | Kaiser Permanente Southern California May 4, 2024 | Kaiser Permanente Northwest April 11, 2024

§Start dates based on the date nirsevimab was implemented, determined based on the date on or after August 3, 2023, that was 7 days after the first instance of nirsevimab receipt was recorded in the data, regardless of whether it occurred in a child with an encounter for RSV-like illness. Columbia University Irving Medical Center October 19, 2023 | HealthPartners October 16, 2023 | Regenstrief Institute October 8, 2023 | Intermountain Health October 9, 2023 | Kaiser Permanente Southern California October 22, 2023 (date reported by site) | Kaiser Permanente Northwest October 20, 2023

¶Adjusted for age, race and ethnicity, sex, calendar day (days since Oct 8, 2023), and geographic region

**Effectiveness estimates from a model that excluded variables with a high degree of missingness from consideration in the calculation of propensity-to-be-immunized weights were similar: 71% (95% CI: 57%-80%).

[††]In the main analysis, nirsevimab effectiveness among those that received nirsevimab 7-59 days prior to the encounter was 76% (95% CI: 66%-83%) and was 78% (95% CI: 62%-87%) among those that received nirsevimab 60-164 days prior to the encounter.

Supplementary Table 8: Evaluating the effect of including additional covariates in logistic regression models for estimation of nirsevimab product effectiveness against RSV-associated ED encounters among infants in their first RSV season.

Models	Adjusted PE (95% CI)
Main Model: adjusted for age, race and ethnicity, sex, calendar day (days since Oct 8, 2023), and geographic region	77 (69-83)
Alternative Model 1: adjusted for age, race and ethnicity, sex, calendar day (days since Oct 8, 2023), geographic region, and *facility urban/rural classification*	77 (68-83)
Alternative Model 2: adjusted for age, race and ethnicity, sex, calendar day (days since Oct 8, 2023), geographic region, and *Social Vulnerability Index of residence**	77 (69-83)
Alternative Model 3: adjusted for age, race and ethnicity, sex, calendar day (days since Oct 8, 2023), geographic region, and *Medicaid coverage*	77 (69-83)
Alternative Model 4: adjusted for age, race and ethnicity, sex, calendar day (days since Oct 8, 2023), geographic region, and *No. of UMC categories*†	77 (69-83)
Alternative Model 5: adjusted for age, race and ethnicity, sex, calendar day (days since Oct 8, 2023), geographic region, and *presence of respiratory disease*‡	77 (69-83)
Alternative Model 6: adjusted for age, race and ethnicity, sex, calendar day (days since Oct 8, 2023), geographic region, and *presence of non-respiratory disease*§	77 (69-83)
Alternative Model 7: adjusted for age, race and ethnicity, sex, calendar day (days since Oct 8, 2023), geographic region, and *presence of ≥1 high-risk UMC*¶	77 (69-83)
Alternative Model 8: adjusted for age, race and ethnicity, sex, calendar day (days since Oct 8, 2023), geographic region, and *preterm birth***	76 (68-82)

RSV: respiratory syncytial virus | CI: confidence interval | PE: product effectiveness | UMC: underlying medical condition | IMV: invasive mechanical ventilation

*Reflects the quartiles of the distribution of Social Vulnerability Index (SVI) among encounters included in this analysis. SVI is defined based on the census tract of residence. The CDC/ATSDR SVI uses 16 U.S. census variables to determine social vulnerability for each census tract. Higher SVI values correspond to higher social vulnerability, which refers to the potential negative effects on communities caused by external stresses on human health.

[†]UMC categories included pulmonary, cardiovascular, cerebrovascular, musculoskeletal, neurologic, hematologic, endocrine, renal, and gastrointestinal.

[‡]Presence of respiratory disease was defined as presence of International Classification of Disease, 10th Revision (ICD-10) discharge diagnosis codes for: asthma, other chronic lung disease, apnea, chronic lung disease of prematurity, cystic fibrosis, congenital airway abnormality, or reactive airway disease.

[§]Presence of non-respiratory disease was defined as presence of ICD-10 discharge diagnosis code for: cardiovascular disease, cerebrovascular disease, neurological and musculoskeletal disease, hematologic disease, endocrine/metabolic disease, renal disease, gastrointestinal and hepatic disease, clinical obesity, clinical underweight, premature birth, developmental delay, or technology dependence.

[¶]High-risk conditions included ICD-10 discharge diagnosis codes for: chronic lung disease of prematurity, congenital heart disease, Down Syndrome, neurological and/or musculoskeletal conditions, cystic fibrosis, congenital airway abnormality, and reactive airway disease.

**Preterm birth status was derived from ICD-10 discharge diagnosis codes and infant records. If either an ICD-10 code corresponding to preterm birth (P07.*) was listed among the discharge diagnoses as the time of the RLI encounter or the recorded gestational age at birth was <37 weeks, then the infant was considered to have been born preterm. Gestational age at birth was unknown for 2,230 (44%) of RLI ED encounters.

Supplementary Figure 2: Analytic population flow for assessment of first season nirsevimab product effectiveness against RSV-associated hospitalization – VISION, October 8, 2023 – March 31, 2024

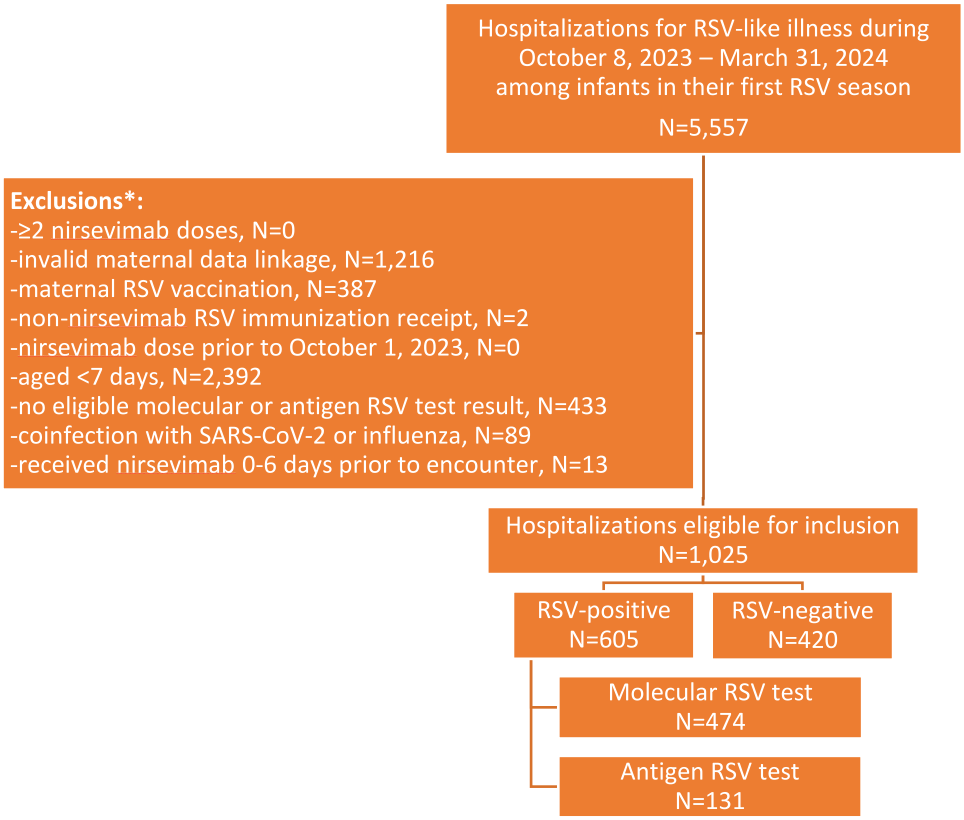

*Exclusions applied in order listed. Some encounters might have met ≥1 exclusion criteria.

Supplementary Table 9. Nirsevimab effectiveness against RSV-associated hospitalization among infants born preterm or with underlying medical conditions, VISION Network, Oct 8, 2023-March 31, 2024

Category | nirsevimab dosage pattern	Total RLI hospitalizations*	RSV-positive hospitalizations N (Row %)	Median days since dose (IQR)	Adjusted PE (95% CI)†
Infants born preterm				
No nirsevimab doses	120	62 (52)	N/A	Ref
Nirsevimab, 7-145 days prior	25	0 (0)	64 (28-84)	98 (84-100)
Infants with ≥1 UMC				
No nirsevimab doses	229	94 (41)	N/A	
Nirsevimab, 7-145 days prior	46	1 (2)	57 (28-78)	95 (82-99)

RSV = respiratory syncytial virus; ED = emergency department; IQR = interquartile range; CI = confidence interval; Ref = reference group; PE = product effectiveness; VISION = Virtual SARS-CoV-2, Influenza, and Other respiratory viruses Network; UMC = underlying medical condition

*Encounters included those among infants in their first RSV season with a diagnosis of RSV-like illness (RLI), excluding infants with evidence of maternal RSV vaccination and infants who received nirsevimab <7 days prior to the index date for the encounter. RLI was defined as ≥1 International Classification of Disease 10th Revision discharge diagnosis code corresponding to one or more of the following: COVID-19 pneumonia, influenza pneumonia, other viral pneumonia, influenza disease, bacterial pneumonia, acute respiratory distress syndrome, asthma exacerbation, respiratory failure, other acute lower respiratory tract infection, sinusitis, acute upper respiratory tract infections, acute respiratory illness signs and symptoms, viral illness not otherwise specified, sepsis, respiratory failure, irritable/fussy infant, respiratory distress of newborn, congenital pneumonia, interstitial emphysema and related conditions, other respiratory conditions originating in the perinatal period, congenital viral diseases, bacterial sepsis of newborn, or other infections specific to the perinatal period.

†PE was calculated as (1 – adjusted odds ratio) x 100%, with adjusted odds ratio calculated using Firth penalized logistic regression, adjusting for age, race and ethnicity, sex, calendar day, and geographic region.

Supplementary Table 10: Sensitivity and secondary analyses results for estimation of nirsevimab product effectiveness against RSV-associated hospitalization among infants in their first RSV season.

Sensitivity/Secondary Analysis	Adjusted PE (95% CI)[¶]
Main Analysis	98 (95-99)
Varying date of eligible encounters based on RSV circulation and/or nirsevimab implementation	
Analytic period: October 8, 2023, through region-specific offset, determined based on NREVSS surveillance data*[†]	98 (94-99)
Analytic period: October 8, 2023, through site-specific offset, determined based on VISION data[‡]	98 (95-99)
Analytic period: Region-specific onset determined based on NREVSS surveillance data*[†] through March 31, 2024	98 (95-99)
Analytic period: region-specific onset and offset, determined based on NREVSS surveillance data*[†]	98 (94-99)
Analytic period: region-specific onset determined based on NREVSS surveillance data*[†] through site-specific offset determined based on VISION data[‡]	98 (95-99)
Analytic period: site-specific onset based on date of nirsevimab implementation[§] through March 31, 2024	98 (95-99)
Analytic period: site-specific onset based on date of nirsevimab implementation[§] through region-specific offset based on NREVSS surveillance data*[†]	98 (94-99)
Restricting to molecular-positive case encounters	
Excluding RSV-antigen positive cases	98 (94-99)
Excluding controls testing positive for SARS-CoV-2 or influenza	
Excluding SARS-CoV-2 and flu-positive controls	98 (95-99)
Including infants with evidence of nirsevimab receipt 0-6 days prior to the encounter	
Including infants with evidence of nirsevimab receipt 0-6 days prior to the encounter	98 (95-99)
Excluding infants with immunocompromising conditions	
Excluding infants with immunocompromising conditions	98 (95-99)
Restricting encounters to those with ARI	
Limiting to ARI encounters	98 (95-99)
Excluding site testing a relatively low proportion of encounters	
Excluding 1 site with relatively low RSV testing	98 (94-99)
Secondary analysis using a weighted multivariable logistic regression model	
Additional adjustment for inverse propensity-to-be-immunized weights	97 (93-99)**

RSV: respiratory syncytial virus | CI: confidence interval | ARI: acute respiratory illness | PE: product effectiveness | NREVSS: National Respiratory and Enteric Virus Surveillance System

*Start and end dates based on the first and last of 2 consecutive weeks when the percentage of PCR tests positive for RSV was ≥3%. HHS Region 2 (Columbia University Irving Medical Center) Start: October

28, 2023, End: January 27, 2024 | HHS Region 5 (HealthPartners and Regenstrief Institute) Start: October 21, 2023, End: March 9, 2024 | HHS Region 8 (Intermountain Health) Start: November 18, 2023, End: April 6, 2024 | HHS Region 9 (Kaiser Permanente Southern California) Start: September 30, 2023 [while this is the onset of the RSV season, eligible encounters occurred on or after October 8, 2023, to coincide with nirsevimab availability in the United States], End: February 24, 2024 | HHS Region 10 (Kaiser Permanente Northwest) Start: October 28, 2023, End: March 2, 2024

[†]https://www.cdc.gov/surveillance/nrevss/index.html

[‡]End dates based on the last observed encounter for RSV-like illness with a positive RSV test. Columbia University Irving Medical Center April 14, 2024 | HealthPartners April 14, 2024 | Regenstrief Institute April 8, 2024 | Intermountain Health April 28, 2024 | Kaiser Permanente Southern California May 4, 2024 | Kaiser Permanente Northwest April 11, 2024

[§]Start dates based on the date nirsevimab was implemented, determined based on the date on or after August 3, 2023, that was 7 days after the first instance of nirsevimab receipt was recorded in the data, regardless of whether it occurred in a child with an encounter for RSV-like illness. Columbia University Irving Medical Center October 19, 2023 | HealthPartners October 16, 2023 | Regenstrief Institute October 8, 2023 | Intermountain Health October 9, 2023 | Kaiser Permanente Southern California October 22, 2023 (date reported by site) | Kaiser Permanente Northwest October 20, 2023

[¶]Adjusted for age, race and ethnicity, sex, calendar day (days since Oct 8, 2023), and geographic region

**Effectiveness estimates form a model that excluded variables with a high degree of missingness from consideration in the calculation of propensity-to-be-immunized weights were similar: 97% (95% CI: 92%-99%).

Supplementary Table 11: Evaluating the effect of including additional covariates in logistic regression models for estimation of nirsevimab product effectiveness against RSV-associated hospitalization among infants in their first RSV season.

Models	Adjusted PE (95% CI)
Main Model: adjusted for age, race and ethnicity, sex, calendar day (days since Oct 8, 2023), and geographic region	98 (95-99)
Alternative Model 1: adjusted for age, race and ethnicity, sex, calendar day (days since Oct 8, 2023), geographic region, and *facility urban/rural classification*	98 (95-99)
Alternative Model 2: adjusted for age, race and ethnicity, sex, calendar day (days since Oct 8, 2023), geographic region, and *Social Vulnerability Index of residence**	98 (94-99)
Alternative Model 3: adjusted for age, race and ethnicity, sex, calendar day (days since Oct 8, 2023), geographic region, and *Medicaid coverage*	98 (95-99)
Alternative Model 4: adjusted for age, race and ethnicity, sex, calendar day (days since Oct 8, 2023), geographic region, and *No. of UMC categories*[†]	98 (94-99)
Alternative Model 5: adjusted for age, race and ethnicity, sex, calendar day (days since Oct 8, 2023), geographic region, and *presence of respiratory disease*[‡]	98 (94-99)
Alternative Model 6: adjusted for age, race and ethnicity, sex, calendar day (days since Oct 8, 2023), geographic region, and *presence of non-respiratory disease*[§]	98 (94-99)
Alternative Model 7: adjusted for age, race and ethnicity, sex, calendar day (days since Oct 8, 2023), geographic region, and *presence of ≥1 high-risk UMC*[¶]	98 (94-99)
Alternative Model 8: adjusted for age, race and ethnicity, sex, calendar day (days since Oct 8, 2023), geographic region, and *preterm birth***	98 (94-99)

RSV: respiratory syncytial virus | CI: confidence interval | PE: product effectiveness | UMC: underlying medical condition | IMV: invasive mechanical ventilation

*Reflects the quartiles of the distribution of Social Vulnerability Index (SVI) among encounters included in this analysis. SVI is defined based on the census tract of residence. The CDC/ATSDR SVI uses 16 U.S. census variables to determine social vulnerability for each census tract. Higher SVI values correspond to higher social vulnerability, which refers to the potential negative effects on communities caused by external stresses on human health.

[†]UMC categories included pulmonary, cardiovascular, cerebrovascular, musculoskeletal, neurologic, hematologic, endocrine, renal, and gastrointestinal.

[‡]Presence of respiratory disease was defined as presence of International Classification of Disease, 10th Revision (ICD-10) discharge diagnosis codes for: asthma, other chronic lung disease, apnea, chronic lung disease of prematurity, cystic fibrosis, congenital airway abnormality, or reactive airway disease.

[§]Presence of non-respiratory disease was defined as presence of ICD-10 discharge diagnosis code for: cardiovascular disease, cerebrovascular disease, neurological and musculoskeletal disease, hematologic disease, endocrine/metabolic disease, renal disease, gastrointestinal and hepatic disease, clinical obesity, clinical underweight, premature birth, developmental delay, or technology dependence.

[¶]High-risk conditions included ICD-10 discharge diagnosis codes for: chronic lung disease of prematurity, congenital heart disease, Down Syndrome, neurological and/or musculoskeletal conditions, cystic fibrosis, congenital airway abnormality, and reactive airway disease.

**Preterm birth status was derived from ICD-10 discharge diagnosis codes and infant records. If either an ICD-10 code corresponding to preterm birth (P07.*) was listed among the discharge diagnoses as the time of the RLI encounter or the recorded gestational age at birth was <37 weeks, then the infant was considered to have been born preterm. Gestational age at birth was unknown for 381 (37%) of RLI hospitalizations.
